# *Pseudomonas aeruginosa* Phage Cocktails: Rational Design and Efficacy Against Mouse Wound and Systemic Infection

**DOI:** 10.3390/antibiotics15010075

**Published:** 2026-01-09

**Authors:** Mikeljon P. Nikolich, Anna C. Jacobs, Tracey L. Peters, Yonas A. Alamneh, Kirill V. Sergueev, Nino Mzhavia, Chaselynn M. Watters, Helen R. Freyberger, Olga A. Kirillina, Emily Engeman, Brett E. Swierczewski, Mark P. Simons, Schroeder M. Noble, Damon W. Ellison, Andrey A. Filippov

**Affiliations:** 1 Wound Infections Department, Bacterial Diseases Branch, Walter Reed Army Institute of Research, Silver Spring, MD 20910, USA; mikeljon.p.nikolich.civ@health.mil (M.P.N.); jacobs.anna.c@gmail.com (A.C.J.); yonas.a.alamneh.civ@health.mil (Y.A.A.); kirill.sergueev@gmail.com (K.V.S.); nino.mzhavia.ctr@health.mil (N.M.); hrfreyberger@gmail.com (H.R.F.); olga.a.kirillina.ctr@health.mil (O.A.K.); emily.engeman.ctr@health.mil (E.E.); schroeder.m.noble.civ@health.mil (S.M.N.); 2 Department of Entomology, Washington State University, Pullman, WA 99164, USA; tracey.peters@wsu.edu; 3 Infectious Diseases Directorate, Naval Medical Research Command, Silver Spring, MD 20910, USA; chaselynn.m.watters.mil@health.mil (C.M.W.); mark.p.simons.mil@health.mil (M.P.S.); 4 Bacterial Diseases Branch, Walter Reed Army Institute of Research, Silver Spring, MD 20910, USA; brett.e.swierczewski.mil@health.mil (B.E.S.); damon.w.ellison.mil@health.mil (D.W.E.)

**Keywords:** *Pseudomonas aeruginosa*, multidrug resistance, phage diversity, phage host range, anti-biofilm effect, phage receptors, phage resistance, phage compatibility, cocktail formulation, phage therapy, mouse wound model, phage treatment efficacy

## Abstract

**Background/Objectives:** Phages show efficacy against multidrug-resistant *Pseudomonas aeruginosa*, but limited host ranges require combining them in cocktails. In this work, we characterized 25 *P. aeruginosa* phages, developed therapeutic cocktails active against diverse clinical isolates, and tested phage efficacy in a mouse incisional wound model. **Methods/Results:** These phages represent seven genera, and genomic and phenotypic analyses indicate that 24/25 are lytic and suitable for phage therapy. Phage host ranges on a diversity panel of 156 *P. aeruginosa* strains that included 106 sequence types varied from 8% to 54%, and together the 24 lytic phages were active against 133 strains (85%). All of the phages reduced bacterial counts in biofilms. A cocktail of five lytic phages, WRAIR_PAM1, covered 56% of the strain panel, protected 100% of mice from lethal systemic infection (vs. 20% survival in the saline-treated group), and accelerated healing of infected wounds. An improved five-phage cocktail, WRAIR_PAM2, was formulated by a rational design approach (using phages with broader host ranges, more complementing activity, relatively low resistance background, and compatibility in mixes). **Conclusions:** WRAIR_PAM2 covered 76% of highly diverse clinical isolates and demonstrated significant efficacy against topical and systemic *P. aeruginosa* infection, indicating that it is a promising therapeutic candidate.

## 1. Introduction

*Pseudomonas aeruginosa* is a ubiquitous and globally spread Gram-negative bacterium and important opportunistic human pathogen that causes >7% of nosocomial infections [1,2]. *P. aeruginosa* is a major causative agent of soft tissue infections including burns [3,4,5,6,7] and acute traumatic [8,9,10,11] and chronic wounds [12,13,14,15]. These infections are typically difficult to treat because of *P. aeruginosa* frequent multidrug resistance [3,9,11,16] and robust biofilm formation associated with enhanced antimicrobial tolerance and wound infection persistence [6,9,12,14,17,18]. The common occurrence of multidrug-resistant (MDR), extensively drug-resistant (XDR) or even pandrug-resistant (PDR) *P. aeruginosa* infections leaves clinicians rapidly diminishing options for antibiotic therapy, which warrants the urgent development of new antimicrobials to augment antibiotics [2,16,19].

A promising class of alternative antibacterials is lytic bacteriophages (phages) [20,21,22,23,24,25]. Phages are capable of dispersing *P. aeruginosa* biofilms and killing bacteria in biofilms [26,27,28]. A number of review articles have described significant efficacy of lytic phages against *P. aeruginosa* infections in different animal models [2,29,30,31,32,33]. Notable efficacy of phages has been shown against *P. aeruginosa* infections of acute traumatic [34,35] and burn [36,37,38] wounds in mice, incisional wounds in rats [39] and rabbits [40], as well as chronic diabetic foot infection in rats [41].

Several clinical cases indicated the efficacy of compassionate phage treatment against *P. aeruginosa* infections. For example, a phage cocktail applied intravenously had a remarkable therapeutic effect in a patient with septicemia caused by an XDR strain of *P. aeruginosa* [42]. Phage in combination with ceftazidime completely cured chronic *P. aeruginosa* infection of an aortic Dacron graft [43]. While antibiotics alone were not effective, positive outcomes of phage treatment combined with antibiotics were observed in patients with severe or refractory XDR *P. aeruginosa* infections, including pneumonia, recurrent bacteremia [44], intravascular stent [45] and left ventricular assist device driveline infection [46,47], osteomyelitis in different bones, mastoiditis, otitis media, sinusitis, and joint and prosthetic joint infections [47].

However, human clinical trials testing phage therapeutics against *P. aeruginosa* infections have shown more modest results. For example, single-dose phage therapy for chronic otitis caused by drug-resistant *P. aeruginosa* with a phage cocktail showed safety but resulted in significant clinical improvement and reduction in bacterial burden in only 6/12 patients, and only three patients had bacterial burden under the detectable level by day 42 [48]. A double-blind, randomized Phase 1/2 trial called PhagoBurn compared the efficacy of topical application of a 12-phage cocktail with standard-of-care (SOC: 1% sulfadiazine silver emulsion cream) treatment against *P. aeruginosa* infection of burn wounds [49]. In this study, participants in the phage treatment group were less extensively burned and older than those in the SOC group. Prior to the study, only two of the 12 phages were tested for stability in storage, and none of the phages were evaluated for compatibility in mixes. Instability of the cocktail drug product appeared to cause a drop in phage titer by seven logs, from 10^9^ to 10^2^ plaque-forming units (PFU) per mL, from the time of manufacture to the time of application. Despite this significant reduction in potency, there was still a reduction in bacterial burden in 9/17 (53%) of subjects in the phage-treated group, compared with 13/17 (76%) of subjects in the SOC-treated group [49]. Eight human clinical trials that involved 9–96 patients treated with phages against chronic otitis, skin ulcers and wounds, septicemia, and systemic infections caused by *P. aeruginosa* produced mixed results that varied from trial to trial and from case to case. While phage therapy demonstrated efficacy in most of the cases in five trials, two trials showed no evidence of a therapeutic effect, and in one trial phage efficacy was lower than that of a SOC antibiotic [22]. There are still no FDA-licensed phage therapeutics in the USA [50].

Most *P. aeruginosa* phages have relatively limited host ranges, with up to 50% of strains susceptible to a single phage [51,52,53,54,55,56,57,58]. Of course, measurements of host range are dependent upon the diversity of the strain panel used for testing. Some phages reported with broader activity were tested against small *P. aeruginosa* strain panels [26,39,59,60,61,62,63] and/or bacterial isolates with limited or undefined diversity [39,59,60,63,64,65,66], e.g., isolated from the same hospital [63,65]. Moreover, *P. aeruginosa* commonly yields phage-resistant mutants, sometimes at high rates [58,65,66,67,68,69,70,71,72,73,74]. Therefore, therapeutic phage mixes (cocktails) need to be developed to cover the majority of clinical isolates and to limit the emergence of phage resistance in the target pathogen [26,35,36,49,55,63,66,67,75,76,77,78,79]. The purpose of this work was to characterize a panel of diverse *P. aeruginosa* phages, develop phage cocktails with broad activity against MDR clinical isolates, and evaluate their therapeutic efficacy against *P. aeruginosa* wound infection in mice. We developed two five-phage cocktails that showed efficacy against topical and systemic infection in mice. One of these cocktails, formulated by rational design (using phages with broader and complementary host ranges, compatible in the mix and binding to different host receptors associated with virulence) showed broader coverage, 76% (119/156) of highly diverse *P. aeruginosa* strains.

## 2. Results

### 2.1. Phage Diversity, Lifestyles, and Morphology

**Figure 1:**
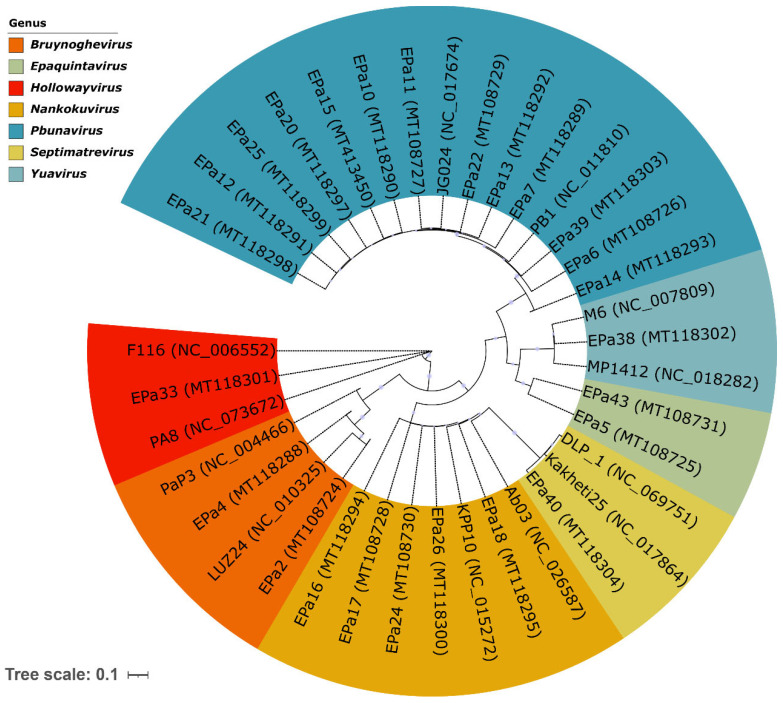
Phylogenetic tree demonstrating genetic diversity and genus designation of 25 *P. aeruginosa* phages, with a predominance of *Pbunavirus* phages. Each genus cluster contains two representative reference phages, selected from the ICTV master species list 2025, except the recently established genus *Epaquintavirus* that was named after EPa5. The tree was constructed by whole genome comparison using VICTOR pipeline [84] with the D4 formula and visualized with iTOL [85].

**Figure 2:**
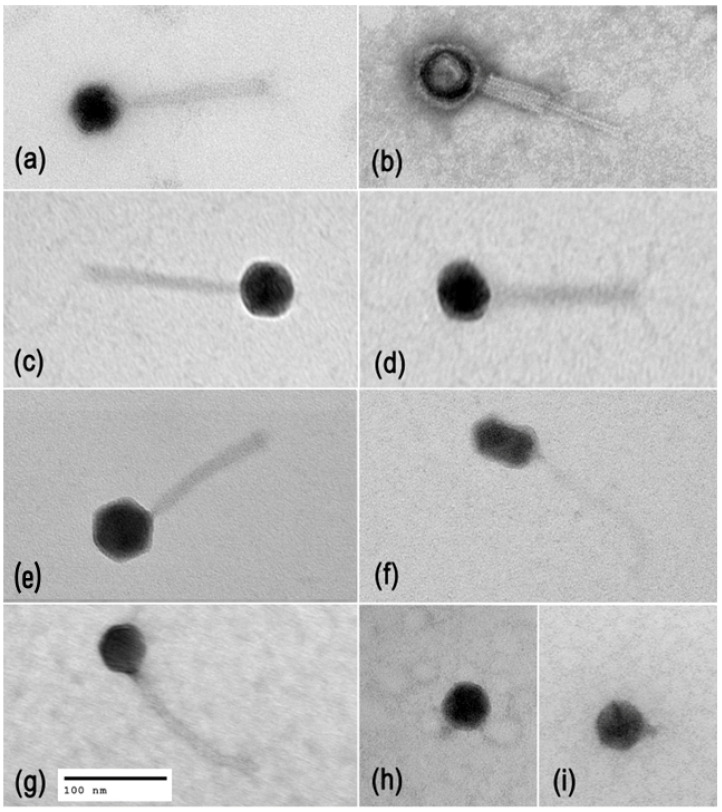
Transmission electron microscopy images of *P. aeruginosa* phage particles. Myovirus morphology: (**a**) EPa6, (**b**) EPa7, (**c**) EPa10, (**d**) EPa13 (genus *Pbunavirus*), and (**e**) EPa18 (genus *Nankokuvirus*). Siphovirus morphology: (**f**) EPa5 (genus *Epaquintavirus*) and (**g**) EPa40 (genus *Septimatrevirus*). Podovirus morphology: (**h**) EPa2 and (**i**) EPa4 (genus *Bruynoghevirus*). The scale bar is common for panels (**a**–**i**).

Twenty-five *P. aeruginosa* phages (Table 1) were previously isolated, and their genomes were sequenced and published as genome announcements [80,81]. Here, we verified phage taxonomy and lifestyles. Mash alignment [82] against the INPHARED database [83] and verification in the ICTV Taxonomy Browser, https://ictv.global/taxonomy, MSL39.v4 (accessed on 10 November 2025) allowed classification of the phages into seven genera, including myophages (*Pbunavirus* and *Nankokuvirus*), siphophages (*Epaquintavirus*, *Yuavirus*, and *Septimatrevirus*), and podophages (*Bruynoghevirus* and *Hollowayvirus*) (Table 1, Figure 1). The first four genera represent three recently established families (*Lindbergviridae*, *Vandenendeviridae*, and *Mesyanzhinovviridae*), while *Septimatrevirus*, *Bruynoghevirus*, and *Hollowayvirus* phages are currently unclassified at the family level. Phage virion morphology was studied by transmission electron microscopy (TEM), which confirmed what the genomic similarities indicated. Typical images of phage virions are shown in Figure 2.

Phage lifestyle predicting classifier BACPHLIP [86] v0.9.6 scored 24 of 25 phages between 75.00% (EPa43) and 99.95% (EPa4), suggesting a virulent lifestyle. The only exception was EPa33, which scored 7.50%, a representative of the genus *Hollowayvirus*, which includes a number of lysogenic phages similar to F116, a generalized transducer [87]. BLASTn analysis of EPa33 genome against the standard nr/nt database at https://blast.ncbi.nlm.nih.gov (accessed on 10 November 2025) displayed many regions of up to 99% identity to *P. aeruginosa* chromosomal DNA, specifically, to entire or partial prophage genomes, suggesting that EPa33 is a lysogenic phage and a potential transducer that cannot be used for therapy. For all of the other phages, no significant homology with bacterial DNA was found, and no significant homology was observed between putative proteins of these 24 phages and products associated with lysogenicity, gene transfer, or bacterial proteins, including antibiotic resistance determinants [88] and virulence factors [89]. Therefore, all phages characterized in this work except *Hollowayvirus* phage EPa33 appear to be lytic and safe for therapeutic use.

Most of the phages were isolated from three different sewage fractions collected on the same day. Exceptions were EPa38, EPa39, and EPa43 obtained from lake water, and EPa40 that was isolated from soil. The most prevalent isolates belonged to the genus *Pbunavirus*: 13/25 (52%), see Table 1 and Figure 1.

### 2.2. Phage Host Range Testing Against Highly Diverse P. aeruginosa Strains

Phage host ranges were tested by plaque assays against a diversity panel of 156 *P. aeruginosa* strains (Table S1), which included mostly drug-resistant isolates from multiple military hospitals located in different U.S. states, Guam, and Afghanistan. The sources of strains included various specimens from patients and hospital environmental swabs (Table S2). The strains were kindly provided by the Multidrug-Resistant Organism Repository and Surveillance Network (MRSN) at Walter Reed Army Institute of Research (WRAIR). The overall panel was established using a core genome multilocus sequence typing (MLST) approach and contains 106 sequence types (STs). This larger panel includes a published 100-strain diversity panel recommended for research and testing new drugs [90] (highlighted in blue in Table S2) that represents 91 STs and 71 distinct drug resistance profiles, including high-risk and epidemic lineages widely spread in the U.S. and globally: ST235, ST111, ST244, ST357, ST175, and ST654 [91,92,93,94]. All of the phages produced clear plaques on at least some of the strains. Phage host ranges varied from 7.7% (EPa38) to 54.5% (EPa15) (Table 1 and Table S1). Altogether, the 24 lytic phages were active against 133/156 (85.3%) of the strains, and 23 *P. aeruginosa* strains were resistant to all 25 phages.

### 2.3. Phage Anti-Biofilm Activity

**Figure 3:**
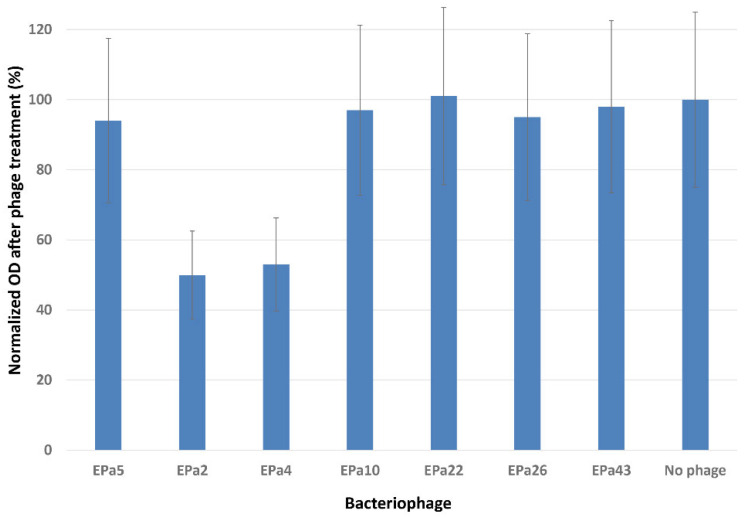
Partial dispersal of *P. aeruginosa* biofilms by phages EPa2 and EPa4 in a crystal violet assay.

**Figure 4:**
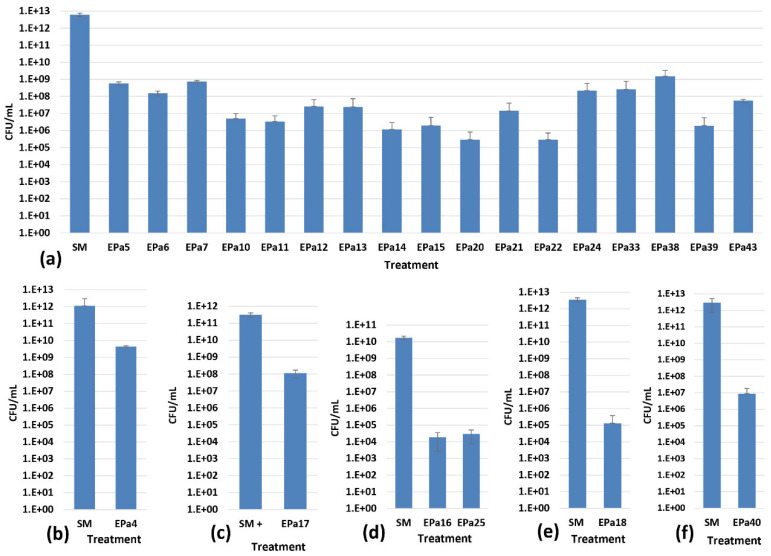
Bactericidal effect of phages against *P. aeruginosa* biofilms. Average colony-forming units per mL (CFU/mL) in biofilms formed by strains (**a**) PAO1::*lux*, (**b**) MRSN 1899, (**c**) MRSN 1409, (**d**) MRSN 1680, (**e**) MRSN 8130, and (**f**) ATCC 10145. SM: SM buffer without phage (negative control).

The ability of phages to degrade biofilm was tested using the crystal violet method with a 96-well MBEC inoculator, as described in Materials and Methods. These experiments performed in sextuplicate showed that only two phages, EPa2 and EPa4, partially dispersed *P. aeruginosa* biofilms (Table 1, Figure 3). Visualization of phage plaque morphology using a dark-field colony counter showed that each of the 25 phages produced plaques with halos, which are indicative of phage polysaccharide depolymerase and anti-biofilm activity [95]. However, the width and intensity of halos varied and depended upon the *P. aeruginosa* strain used. This might suggest an underestimation of the number of phages with anti-biofilm activity. Therefore, bactericidal activity of each phage in *P. aeruginosa* biofilms was assessed using six *P. aeruginosa* strains that were permissive for different phages, MRSN 1409 (for EPa17), MRSN 1680 (for EPa16 and EPa25), MRSN 1899 (for EPa2 and EPa4), MRSN 8130 (for EPa18), ATCC 10,145 (for EPa40), and PAO1::*lux* (for all other phages). The results demonstrated that all phages effectively kill *P. aeruginosa* in biofilms, reducing bacterial counts by 2–7 logs (Figure 4).

The high counts of *P. aeruginosa* (n × 10^12^ CFU/mL) observed in our 42 h biofilms have been previously reported by others for several bacterial species grown under different conditions [96,97,98,99,100,101,102,103,104,105].

### 2.4. Phage Cocktail WRAIR_PAM1 and Its Efficacy in a Mouse Wound Model

**Figure 5:**
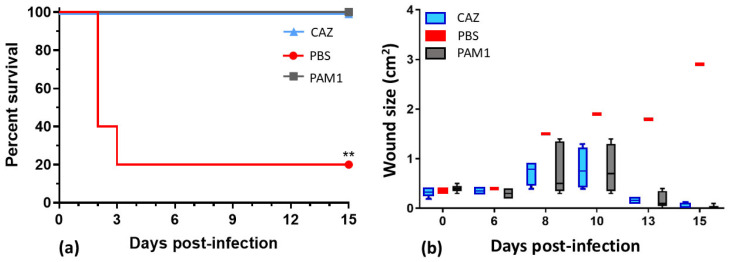
Efficacy of phage cocktail WRAIR_PAM1 (PAM1) against *P. aeruginosa* topical and systemic infection in mice: (**a**) mouse survival and (**b**) wound healing dynamics. CAZ: mice treated with standard-of-care antibiotic ceftazidime (positive control); PBS: mice treated with phosphate-buffered saline (negative control); PAM1: mice treated with five-phage cocktail. Mouse groups were compared for survival using Gehan–Breslow–Wilcoxon test. ** *p* = 0.0046. N = 5 for each mouse group.

The first iteration of *P. aeruginosa* therapeutic five-phage cocktail, WRAIR_PAM1 (PAM1), was formulated before all the 25 phages were fully characterized. Its composition was based mainly on phage anti-biofilm activity (along with their strictly lytic nature, diversity, and, to some extent, on host range data). In addition to phages EPa2 and EPa4 that showed some biofilm dispersal activity (Figure 3), PAM1 included EPa5, EPa6, and EPa17 that were first tested for killing *P. aeruginosa* in biofilms and demonstrated a bactericidal effect. This cocktail was lytic against 87/156 strains and thus covered 55.8% of the diversity panel. PAM1 was tested for efficacy in a mouse dorsal full-thickness wound infection model (Figure 5). Three groups of five BALB/c mice each were challenged with *P. aeruginosa* strain PAO1::*lux* and treated for five days. Group 1 was treated with 10^9^ PFU of PAM1 (2 × 10^8^ for each cocktail component) once a day, both topically and intraperitoneally (IP). Group 2 was treated with phosphate-buffered saline (PBS) once a day, topically and IP (negative control). Group 3 was treated with standard-of-care antibiotic ceftazidime (CAZ, positive control, given only IP twice a day). Both PAM1 and CAZ protected 100% of mice from lethal systemic *P. aeruginosa* infection compared to 20% survival in the negative control group treated with PBS (see Figure 5a) and accelerated healing of infected dorsal wounds. Wound area in phage-treated and ceftazidime-treated mice at day 16 was 0.0 cm^2^ vs. a necrotizing, expanding wound of 2.9 cm^2^ in the PBS-treated, surviving mouse (Figure 5b). Bacterial burden at day 15 in phage- and antibiotic-treated mice was below the limit of detection (10^4^ photons/second) compared to 10^6^ in the PBS-treated survivor.

### 2.5. Using Rational Design for Developing an Improved Phage Cocktail

The purpose of further experiments was to develop an improved therapeutic phage cocktail with broader coverage of MDR *P. aeruginosa* clinical isolates and low bacterial resistance. We endeavored to use diverse, stable, strictly lytic phages with broader, more complementary host ranges and anti-biofilm activity, that bind to different receptors (such as virulence determinants, to promote emergent phage-resistant phenotypes with potential virulence attenuation trade-offs), have relatively low resistance frequencies, and do not show antagonism to each other in mixes, so as to provide high efficacy of the new cocktail in vivo.

### 2.6. Identification of Phage Receptors on Bacterial Cell Surface

Panels of *P. aeruginosa* PAO1 knockout mutants with defects in the biosynthesis of different LPS components, type IV pili, and flagella were used for plaque assays to identify phage host receptors (Table S3). All of the phages lytic against the strain PAO1 required type IV pili (T4P), but differed in their secondary binding receptor, utilizing various LPS moieties, including core, A, or B bands, especially long and very long B bands (Table 1 and Table S2).

### 2.7. Predicted Pairwise Host Ranges and Selection of Promising Phage Candidates

Lytic phages of five genera (*Pbunavirus*, *Nankokuvirus*, *Bruynoghevirus*, *Epaquintavirus*, and *Septimatrevirus*) except EPa5 and EPa25 that had relatively narrow lytic spectra (see Table 1 and Table S1) were analyzed for predicted host range complementarity in pairs. Seven phages (EPa11, EPa16, EPa17, EPa22, EPa24, EPa40, and EPa43) appeared to have good complementarity in all 21 pairwise combinations, with predicted broader activity in mixes than as individual phages (Table 2). These seven phages, which belong to four different genera, have relatively broad host ranges, and bind to at least four different receptors (Table 1), were selected for further characterization as promising candidates for a new phage cocktail with expanded activity. The mix of these seven phages (Mix-7) covered 78.2% of the *P. aeruginosa* strain panel, and a mix of five of them (EPa11, EPa17, EPa22, EPa24, and EPa43, Mix-5) covered 76.3% of the strains (Table 2).

### 2.8. Testing Stability of Single Phages and Their Pairs

The PhagoBurn clinical trial suggested instability of some *P. aeruginosa* phages in mixes [49]. Therefore, the seven selected phages and their pairwise combinations were tested for stability in storage at 4 °C. No significant reduction in viability of each of the seven phages and their 21 pairwise mixes was observed for nine months (Figure S1). Moderate fluctuations of the titers were within the error of the method.

### 2.9. Measuring Frequencies of Host Resistance Mutations

Determination of frequencies of *P. aeruginosa* resistance mutations towards each of the seven phages and their pairwise combinations confirmed the lack of phage–phage antagonism (Table 3). Moreover, combinations of phage EPa17 with EPa11, EPa22, EPa24, and EPa40 provided roughly 1-log lower phage resistance frequencies than expected based on resistance frequencies to individual phages. Resistance frequencies to single phages varied approximately from 10^−5^ to 10^−8^. EPa43 demonstrated the lowest resistance frequency that was also observed in all pairwise mixes with this phage. Much slower growth of phage-resistant mutants of *P. aeruginosa* in comparison with the parental strains was observed. This is another indication of the poor fitness of phage-resistant mutants. Four phages out of the seven (EPa16, EPa17, EPa40, and EPa43) had relatively narrow host ranges. EPa16 and EPa40 did not show advantage over other phages in host resistance levels and were excluded from candidates for a new cocktail. EPa43 demonstrated the lowest frequency of *P. aeruginosa* resistance, and EPa17 exhibited fairly low levels of *P. aeruginosa* resistance and reduced the frequencies of resistance in pairs with other phages, so EPa17 and EPa43 were selected for an improved phage cocktail.

### 2.10. Improved Cocktail WRAIR_PAM2 and Its Efficacy Against P. aeruginosa Infection in Mice

**Figure 6:**
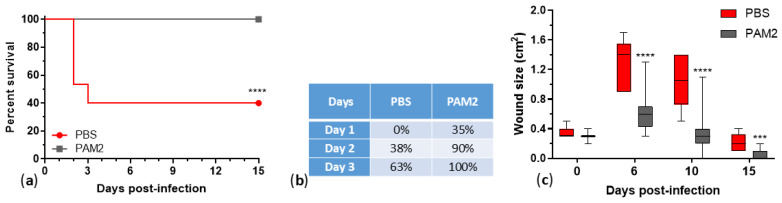
Efficacy of phage cocktail WRAIR_PAM2 (PAM2) against mouse topical and generalized *P. aeruginosa* infection: (**a**) mouse survival (mouse groups were compared using Gehan–Breslow–Wilcoxon test, **** *p* < 0.0001); (**b**) comparison of mouse nest building activity; and (**c**) wound healing dynamics (groups were compared at each time point using Mann–Whitney *t*-test, **** *p* < 0.0001, *** *p* < 0.001). PBS, mice treated with placebo, phosphate-buffered saline (negative control); PAM2, mice treated with the phage cocktail. N = 15 for PBS-treated mouse group; N = 20 for phage-treated group.

A new iteration of a five-phage cocktail, WRAIR_PAM2 (PAM2: EPa11, EPa17, EPa22, EPa24, and EPa43, see Table 4), was formulated by rational design (using diverse, stable, strictly lytic phages with broader and complementary host ranges, anti-biofilm activity, and binding with at least three different receptors in virulent factors, with relatively low bacterial resistance frequencies and compatible in the mix). PAM2 covered 76.3% of diverse MDR clinical isolates (see Mix-5 in Table 2) and demonstrated 100% survival and significant acceleration of wound size healing in PAM2-treated mice compared to the saline-treated group (Figure 6), indicating that this cocktail is a promising therapeutic candidate. Table 4 shows the difference between cocktails PAM1 and PAM2.

## 3. Discussion

*P. aeruginosa* is a ubiquitous and non-fastidious bacterium prone to multidrug resistance and robust biofilm formation and one of the most significant causes of hospital-acquired infections, including traumatic and burn wound infections [1,2,3,4,5,7,10,11,12,15,16,17,18,19]. Even though phages have been shown to be effective antibacterials for the treatment of experimentally infected animals, as well as humans with severe MDR *P. aeruginosa* infections via the expanded access approach, there is still no FDA-licensed phage therapeutic in the USA [20,21,22,23,24,25,50]. The purpose of this work was to characterize a panel of lytic phages and design and preclinically test a fixed phage cocktail with broad activity against diverse MDR clinical isolates of *P. aeruginosa*.

Twenty-five *P. aeruginosa* phages isolated previously [80,81] were characterized here for virion morphology, taxonomy, lifestyles, stability, host ranges, complementarity, anti-biofilm activity, receptors, resistance frequencies, and compatibility. They included myo-, sipho-, and podophages (Figure 2) and belonged to seven genera (Table 1, Figure 1), of which the representatives of *Pbunavirus*, *Nankokuvirus*, *Epaquintavirus*, *Septimatrevirus*, *Yuavirus*, and *Bruynoghevirus* had relatively high BACPHLIP [86] virulence scores, did not contain any putative determinants of transduction, nor showed significant identity to bacterial genomes, and thus were considered lytic and safe for treatment purposes. Data suggesting lytic lifestyles of *Pbunavirus* [55,60,65,80,81,106], *Nankokuvirus* [55,80,81,107], *Septimatrevirus* [64,81], and *Bruynoghevirus* [52,55,80,81] phages have been reported previously. Siphophages EPa5, EPa38, and EPa43 belong to the family *Mesyanzhinovviridae* (Table 1), the representatives of which are sometimes thought to be temperate based on plaque morphology, lytic potential, and misannotation of their genes as integrase and repressor genes [61,108,109,110,111]. However, *Mesyanzhinovviridae* phages demonstrated only lytic cycle infection in *P. aeruginosa* specifically and failed to produce stable lysogenic strains [109,112,113]. Their genomes lacked bacterial DNA, integrase, excisionase, or recombinase genes. The putative “integrase” described before was later identified as a DNA primase/helicase, and the repressor seems to play a role unrelated to lysogeny [80,81,112,113]. Thus, *Epaquintavirus* phages EPa5 and EPa43 and *Yuavirus* phage EPa38 (Table 1, Figure 1) also appear to be lytic. Only *Hollowayvirus* phage EPa33 was considered lysogenic, based on the very low BACPHLIP score (7.5%), homology to *Pseudomonas* DNA, presence of putative integrase and repressor genes in its genome, and high whole genome identity to F116 [85], MD8 [114], and other lysogenic phages from this genus. Thus, EPa33 cannot be used for phage therapy.

The 25-phage panel was tested for host ranges against 156 highly diverse *P. aeruginosa* strains, mostly drug-resistant (136/156, including MDR, XDR, and PDR) isolates from multiple hospitals located in different U.S. states, Guam, and Afghanistan. The sources of strains included various specimens from patients (swabs from traumatic, burn, and surgical wounds, abscesses, respiratory, urine, blood, cerebrospinal fluid, and tissue samples), as well as hospital environmental swabs (Table S2). The MLST-based diversity strain panel contains 104 known STs and two novel STs, combinations of alleles not currently known in the PubMLST database (https://pubmlst.org). These 156 strains include a highly diverse 100-strain panel previously recommended for basic and applied research [90]. The 100-strain panel (highlighted in blue in Table S2) represents 91 STs and 71 distinct antibiotic susceptibility profiles, including high-risk MDR/XDR/PDR and global epidemic lineages ST235, ST111, ST244, ST357, ST175, and ST654 [91,92,93,94]. Using such diversity strain panels is important for the development of broad-range therapeutic phage cocktails active against the majority of MDR clinical isolates circulating in a certain area, country, or even globally.

Phage host ranges varied from 7.7% to 54.5% (Table 1 and Table S1). Given the high diversity of *P. aeruginosa* strains tested, coverage of ≥30% by any single phage was considered a broad host range. Several publications describing *P. aeruginosa* phages with host ranges significantly greater than 50% used small strain panels [26,39,59,60,61,62,63] and/or bacterial isolates with limited or undefined diversity [39,59,60,63,64,65,66], for example, isolated from one hospital [63,65]. Overall, both the entire 25-phage panel and 24 lytic phages tested in this work were active against 133/156 (85.3%) of the strains, with only 23 strains resistant to all 25 phages. All these 23 strains were drug-resistant and a potential correlation with sequence types was observed: 2/2 ST17, 3/6 ST621, and 4/11 ST235 strains were pan-phage-resistant (Table S2). Correlation of ST235 with broader phage resistance has been noted by our team previously [115].

All 25 phages displayed bactericidal effects in *P. aeruginosa* biofilms, reducing live bacterial counts by two to seven logs (Figure 4), and two of them, EPa2 and EPa4, were able to disrupt biofilms (Figure 3). They both belong to the genus *Bruynoghevirus* and are closely related to phage LUZ24, which produces several proteins with anti-biofilm activity [116]. Many other *P. aeruginosa* phages have previously demonstrated different levels of anti-biofilm effects, including bactericidal action and dispersal of biofilm [26,27,28,34,38,58,65,66,75,117]. The first five-phage cocktail designed in this work, PAM1, was based mainly on anti-biofilm activity of its components. This early version of phage cocktail was designed before the full characterization of the 25 phages. Some additional criteria for selection of its components were the phage strictly lytic nature, diversity and, to some extent, host range results. This cocktail was active against 55.8% of diverse *P. aeruginosa* clinical isolates and displayed significant efficacy in a mouse incisional wound model by protecting mice from lethal systemic infection and accelerating wound healing (Figure 5). The efficacy of phage cocktails against *P. aeruginosa* infections of acute traumatic wounds in mice [34,35,118] and rabbits [40] was demonstrated previously, and even single phages effectively killed the bacterium and accelerated wound healing in rats [39,119]. A feature of our animal experiment evaluating the efficacy of phage cocktail PAM1 was the use of a relatively high infectious dose of *P. aeruginosa* PAO1::*lux* that resulted in high mortality in the control saline-treated mice.

Rational design of durable fixed therapeutic phage cocktails includes the use of diverse, stable, strictly lytic phages with safe genomic properties (no transduction determinants and bacterial DNA), with broad and complementary host ranges tested in strain diversity panels, demonstrating potent anti-biofilm activity, binding to different receptors, exhibiting relatively low host resistance rates, compatible in mixes with other phages, and showing efficacy in vivo using different animal models [21,66,74,75,76,77,78,79]. In this work, we used all these criteria to develop PAM2, a novel broad-range therapeutic phage cocktail against MDR *P. aeruginosa*.

Five putative receptors for the 25 phages were identified in T4P and different parts of LPS, i.e., all phages representing seven genera utilize T4P and some LPS moiety (ies) for their binding. In addition to T4P, phage receptors may include LPS core, A, or B bands, the most frequent being long and very long B bands (Table 1 and Table S2). There is an extensive history of *P. aeruginosa* LPS and T4P recognition as major phage receptors [120,121]. It is known that LPS is used as a receptor by lytic phages now assigned to the genera *Pbunavirus* [65,121,122,123,124,125], *Septimatrevirus* [126], *Pakpunavirus* [54,69,127], *Luzseptimavirus* [124,128], and a temperate, cytotoxin-converting phage ɸCTX (genus *Citexvirus*) [129]. T4P of *P. aeruginosa* has been described as a receptor for lytic *Yuavirus* [122,123], *Phikmvvirus* [130,131], *Phikzvirus* [124,132], *Abidjanvirus*, *Zicotriavirus* [133], and *Pepevirus* [120,134] phages, as well as temperate phages F116 [87], Pf1 [135], D3112, and B3 [136] that currently belong to the genera *Hollowayvirus*, *Primolicivirus*, *Casadabanvirus*, and *Beetrevirus*, respectively. A jumbo *Wroclawvirus* phage PA5oct required both LPS and T4P for *P. aeruginosa* lysis [58]. Our data suggest a broader simultaneous involvement of *P. aeruginosa* LPS and T4P in phage binding. Both LPS [69,137] and T4P [138] are major *P. aeruginosa* virulence factors. This suggests that receptor-dependent phage-resistant mutants arising during phage therapy would have reduced virulence and thus could be more easily cleared by the immune system.

Temperate phage EPa33 (*Hollowayvirus*) and lytic phages EPa5 (*Epaquintavirus*) and EPa25 (*Pbunavirus*) with relatively narrow lytic spectra were excluded from further analysis. Twenty-two lytic phages of five genera (*Pbunavirus*, *Nankokuvirus*, *Bruynoghevirus*, *Epaquintavirus*, and *Septimatrevirus*) (see Table 1) were analyzed for predicted host range complementarity in pairs. Seven phages (EPa11, EPa16, EPa17, EPa22, EPa24, EPa40, and EPa43) represented four genera (*Pbunavirus*, *Nankokuvirus*, *Epaquintavirus*, and *Septimatrevirus*) bound at least to four different receptors and had relatively broad host ranges. In addition, they complemented each other in all 21 pairwise combinations: the mixes had broader activity than each single phage (see Table 2). These seven phages were selected for further evaluation as likely candidates for an improved phage cocktail.

Phages show different stability in storage, and it is important to maintain phage activity in therapeutic preparations [139]. In addition, a phenomenon termed phage interference can occur during coinfection, as evidenced by reduced burst size of some phages when mixed with another phage [140]. Lytic *P. aeruginosa* phages can compete when, for example, a phage with a shorter lysis time depletes bacteria quickly, reducing the number of host cells for the slower growing phage [141]. An apparent case of phage–phage antagonism was described when mixing 12 lytic phages for the treatment against *P. aeruginosa* in burn wounds resulted in reduction in viable phage in the cocktail by seven orders of magnitude [49]. Thus, we tested the selected seven phages and their 21 pairwise combinations for stability in storage at +4 °C for nine months. No significant reduction in titers of individual phages or their pairwise mixes was evident (Figure S1). However, the existence of unstable phages [25,139] and phage–phage antagonism [49,140,141] makes testing stability of single phages and their mixes an important part of rational design of effective therapeutic cocktails.

There is great variation observed in bacterial resistance rates for different phages in different host bacteria, ranging between 10^−1^ [73] and <10^−10^ [142]. Phages with lower resistance rates are preferable for therapeutic use. We determined the frequencies of *P. aeruginosa* resistance mutations for each of the seven selected phages and their pairwise combinations (Table 3). Resistance frequencies to individual phages varied roughly between 10^−5^ and 10^−8^. Phage EPa43 demonstrated the lowest resistance frequency, a trend that was also observed in all pairwise mixes with this phage. These experiments not only confirmed the lack of phage–phage antagonism in pairs, but even suggested some potential synergy. The combinations of phage EPa17 with EPa11, EPa22, EPa24, and EPa40 demonstrated one log lower resistance frequencies than each single phage with lower resistance from the pair. *P. aeruginosa* phage-resistant mutants displayed very slow growth compared to the parental strains. This confirmed a reduced fitness cost typical for phage-resistant mutants that can be beneficial for therapeutic use of these phages [142,143].

Seven phages (EPa11, EPa16, EPa17, EPa22, EPa24, EPa40, and EPa43) together covered 78.2% of the *P. aeruginosa* strain panel. Among four phages with narrower host ranges, EPa16 and EPa40 were excluded, while EPa17 and EPa43 were included based on the phage resistance criterion: EPa43 demonstrated the lowest frequency of bacterial resistance, and EPa17 showed not only a relatively low level of *P. aeruginosa* resistance, but also reduced the levels of resistance in combination with other phages. EPa11, EPa17, EPa22, EPa24, and EPa43 covered as much as 76.3% of the strains tested (Table 2). These five phages made up the new cocktail, PAM2, that was developed using rational design. The design included exploiting diverse, stable, strictly lytic phages with broader and complementary host ranges, robust anti-biofilm activity, and binding to different host receptors associated with virulence, with relatively low rates of bacterial resistance and compatible in mixes. PAM2 demonstrated efficacy against both local and systemic *P. aeruginosa* infection. Phage treatment rescued 100% of mice from lethal systemic infection, accelerated wound healing and promoted nest building activity (Figure 6).

Therefore, we developed a rational design approach and established a pipeline to produce phage cocktails with broad activity against highly diverse MDR *P. aeruginosa* clinical isolates. The phage cocktail PAM2 developed in this effort is a promising therapeutic candidate. The efficacy of phage cocktails was evaluated using a laboratory antibiotic-susceptible strain of *P. aeruginosa*, PAO1::*lux*. We understand this limitation for testing phage therapeutic products and plan to preclinically test the efficacy of the PAM2 cocktail against a recent high-risk MDR clinical isolate of *P. aeruginosa*.

## 4. Materials and Methods

### 4.1. Bacterial Strains, Media, and Storage of P. aeruginosa and Phage Stocks

A total of 156 *P. aeruginosa* strains were used in this work, mostly multidrug-resistant isolates from military hospitals, were received from the Multidrug-resistant Organism Repository and Surveillance Network (MRSN, WRAIR) and are listed in Supplementary Table S2. Cultures were grown in Heart Infusion Broth (HIB, Becton, Dickinson and Co., Franklin Lakes, NJ, USA) or on plates of 1.5% HIB agar. For phage plating, 0.7% semisolid HIB agar was overlaid on 1.5% HIB agar. *P. aeruginosa* cultures were stored at −80 °C in HIB containing 15% glycerol. Phages were filter sterilized through a 0.22 µm membrane and stored at 4 °C in HIB or sodium chloride–magnesium sulfate (SM) buffer (Teknova, Hollister, CA, USA).

### 4.2. Phage Propagation, Purification, Titration, and Plaque Assays

Phage dilutions were prepared in SM buffer. Phage titration and plaque assays were performed by the double-layer agar method as described earlier [144], with overnight incubation at 37 °C. Phages were propagated on the enrichment strains as follows. Phage stock lysate was added to 150 mL of an early exponential phase bacterial culture grown in HIB at a multiplicity of infection (MOI) of 0.01 and incubated in a 250 mL plastic Erlenmeyer flask at 37 °C at 200 rpm until visible lysis occurred (for 3–5 h). The phage lysate was treated with chloroform added to the final concentration of 5% (*v*/*v*) to destroy all remaining live bacteria. Bacterial debris was removed by 15 min centrifugation at 5000× *g*. The supernatant was filtered through a sterile 0.22 μm membrane. Endotoxin levels in phage suspensions were measured using an Endosafe nexgen-PTS device (Charles River Laboratories, Wilmington, MA, USA). Phage stocks for animal experiments were purified using CsCl gradient centrifugation [144], octanol extraction [145], and chromatography with EndoTrap bulk resin (Hyglos GmbH, Bernried am Starnberger See, Germany), as per the manufacturer’s protocol, to ensure that the endotoxin level was below 500 EU per 10^9^ PFU.

### 4.3. Phage Host Range Testing

Phage host ranges were tested by a modified efficiency of plating assay [146]. Briefly, ten-fold serial dilutions of the phage suspensions were prepared in a sterile flat bottom 96-well plate. Two microliters of each phage dilution ranging from 10^−1^ to 10^−8^ were spotted using a multichannel pipette on semisolid HIB agar overlay infused with *P. aeruginosa* culture and incubated overnight at 37 °C. The results were scored, and the morphology of individual plaques was evaluated. The efficiency of plating (EOP) of phage was calculated as phage titer on the test strain divided into the titer of the same phage on *P. aeruginosa* strain PAO1.

### 4.4. Biofilm Degradation Assay Using an MBEC^TM^ Biofilm Inoculator

Biofilm degradation assays were performed as described [147], with modifications. Briefly, overnight cultures of *P. aeruginosa* were grown in HIB, diluted in a tube with sterile HIB + 10% human serum (Sigma-Aldrich Corporation, St. Louis, MS, USA) to adjust to an approximate concentration of cell density of 100 CFU/mL, vortexed for approximately 10 s and used as an inoculum. One hundred-fifty microliters of the inoculum were placed into each well of the 96-well uncoated polystyrene plate of a MBEC^TM^ Biofilm Inoculator (Innovotech Inc., Edmonton, AB, Canada). The peg lid was placed on the inoculator base, sealed with Parafilm and incubated at 37 °C for 24 h with shaking at 200 rpm on the plate shaker. A series of ten-fold phage dilutions along with no-phage control were prepared in the 96-well plate. Loosely attached cells were removed from the biofilms formed on the pegs by placing the peg lid onto the 96-well plate filled with sterile normal saline for approximately 10 s, the lid with biofilms was transferred into the challenge plate with phage dilutions and incubated at 37 °C for 4 h without shaking. The lid was removed from the incubation plate, rinsed twice in saline, the biofilm on the pegs was fixed in 99% methanol for 15 min, air-dried, and stained with 0.1% crystal violet (CV) in 12% ethanol for 15 min at room temperature. The pegs were washed in tap water to remove unbound CV, air-dried and the dye bound to biofilm was extracted with 200 µL of 100% ethanol in a 96-well plate. The absorbance of the eluted dye was measured at 595 nm on a plate reader. The experiment for each phage was repeated five times and average values of optical density for six experiments were determined.

### 4.5. Phage Bactericidal Activity in Biofilms

Strains were streaked out from glycerol stocks on HIB agar plates two days prior to the experiment. Overnight bacterial cultures were prepared the day before the experiment. Two milliliters of LB broth (Becton, Dickinson and Co.) were added to a 14 mL Falcon tube. A single isolated bacterial colony was picked using a sterile inoculation loop and suspended in LB broth. This culture was allowed to shake (200 rpm) at 37 °C for 14–18 h. To prepare the biofilm culture, overnight cultures were diluted 1:100 by volume in Tryptic Soy Broth (Becton, Dickinson and Co.) with 10% human plasma collected from whole blood in Na heparin (Valley Biomedical Products and Services, Winchester, VI, USA), and 200 µL of diluted culture was added to the interior wells of a 96-well plate. Exterior wells were filled with 200 µL of phosphate-buffered saline (PBS, Sigma-Aldrich Corporation) to counteract the edge effect. Inoculated plate was incubated at 37 °C for 18 h. Phages were diluted down to 5 × 10^9^ PFU/mL, and 50 µL of diluted phage was added to the well (four wells were treated with each phage). SM buffer was used as a control (50 µL in four wells). All treatments were added slowly to the back corner of the plate to avoid disrupting the biofilm. The plate was returned to the incubator for 24 h at 37 °C. After treatment and incubation, four wells from each treatment row were plated for CFU as follows. The biofilm plate was flipped and given a top-down shake to remove the excess media and planktonic bacteria, and the lid was replaced. Two hundred microliters of PBS with 40 mM sodium citrate were added to each well and the biofilm resuspended by pipetting 150 µL 10 times. A deep 96-well plate with 900 µL PBS in each well was prepared. One hundred microliters of sample from each well were transferred to the first row of the deep well plate, mixed five times before continuing to dilute 1:10 down the plate. Five microliters of sample from each well were plated on HIB agar in square grid Petri dishes, in duplicate, and incubated overnight at 37 °C. When determining CFU, the most concentrated dilutions that provided easily countable colonies were used.

### 4.6. Transmission Electron Microscopy of Phage Particles

Phages were prepared for transmission electron microscopy as described previously [148], with modifications. Briefly, phage suspensions were washed twice with 0.1% ammonium acetate by centrifugation for 3 h at 13,250× *g*, phage titers were adjusted to 10^9^ PFU/mL, phage preparations were deposited on 300 mesh carbon-coated copper grids (Electron Microscopy Sciences, Hatfield, PA, USA), stained with 2% uranyl acetate for 1 min, and assessed under a JEM-1400 electron microscope (JEOL Ltd., Tokyo, Japan) at 80 kv. The phage particle images were analyzed with ImageJ software v1.53 (National Institutes of Health, Bethesda, MD, USA).

### 4.7. Phage Receptor Identification Using P. aeruginosa PAO1 Isogenic Mutants

Preliminary mapping of phage receptors was performed by plating phages on two panels of *P. aeruginosa* PAO1 knockout mutants with defects in the synthesis of different parts of LPS, type IV pili, and flagella (Table 5), compared to the parental strain PAO1.

### 4.8. Phage Genome Analysis

The genomes of all 25 *Pseudomonas* phages have previously been sequenced and published [80,81]. Here, these 25 genomes and 12 reference phage genomes were downloaded from NCBI, and phage annotation was conducted using the Pharokka pipeline [149] v 1.7.0, classified using mash [82] v2.2.2 against the INPHARED database [83] and verified using BLASTn+ [150] v2.16.0 alignments and the ICTV Taxonomy Browser (https://ictv.global/taxonomy, MSL40.v1, accessed 10 November 2025). A phylogenetic tree was created using the VICTOR pipeline [84]. Pairwise comparisons of the amino acid sequences were conducted using the Genome-BLAST Distance Phylogeny (GBDP) method [151] under settings recommended for prokaryotic viruses [84]. The resulting intergenomic distances were used to infer a balanced minimum evolution tree with branch support via FASTME 2.0 [152] using the D4 formula. Branch support was inferred from 100 pseudo-bootstrap replicates each. Trees were rooted at the midpoint [153]. The phylogenetic tree was visualized using iTOL v6 [85]. Lifestyle scoring was conducted in BACPHLIP [86]. Protein homology detection was performed using MMseqs2 [154] v18-8cc5c against CARD [88] and VFDB [89] databases, and searches were conducted using HHpred [155] at https://toolkit.tuebingen.mpg.de/tools/hhpred (accessed on 10 November 2025).

### 4.9. Testing Stability of Single Phages and Their Pairs

The seven single CsCl-purified phages EPa11, EPa16, EPa17, EPa22, EPa24, EPa40, and EPa43 were diluted in SM buffer and used in different titers varying from 10^9^ to 7 × 10^10^ PFU/mL, in order to be able to discriminate viability curves in graphs. Similarly, 21 pairwise combinations of these phages were mixed in a ratio of 1:1, total titers ranging between 10^9^ and 8 × 10^10^ PFU/mL. Single phages and pairwise mixes were stored for nine months at 4 °C in 1.8 mL Nunc^TM^ cryogenic tubes (ThermoFisher Scientific Inc., Waltham, MA, USA) wrapped in aluminum foil and plated on the culture of each *P. aeruginosa* strain susceptible to a specific phage) in semisolid HIB agar overlays at 3, 10, 23, 30, and 85 days and 6 and 9 months.

### 4.10. Determination of P. aeruginosa Resistance Mutation Frequencies to Single Phages and Phage Pairs

One hundred microliters of overnight culture of *P. aeruginosa* strain MRSN 321 (susceptible to each of phages EPa11, EPa16, EPa17, EPa22, EPa24, and EPa40) diluted in PBS and containing > 10^8^ CFU were plated on semisolid HIB agar overlays containing 10^9^ PFU of an individual phage, or each of 21 pairwise mixes of these phages (5 × 10^8^ + 5 × 10^8^ PFU). In parallel, the bacterial culture was titrated in PBS down to 10^−6^, the 10^−5^, and 10^−6^ dilutions (100 µL) were plated on HIB agar plates in triplicate. To calculate phage resistance frequencies, CFU counts on phage plates were divided into the counts on HIB agar without phage.

### 4.11. Testing of Phage Cocktail Efficacy in a Mouse Wound P. aeruginosa Infection Model

Treatment with PAM1 and PAM2 was evaluated in our previously developed mouse dorsal wound model [35], with modifications. Briefly, 6–8-week-old female BALB/c mice (18–22 g, substrain BALB/cAnNCr, Charles River Laboratories, Wilmington, MA, USA) received irradiated food and water ad libitum. They were housed in groups in sanitized solid-bottom individually ventilated cages on sterile paper bedding and were provided with environmental enrichment. The animals were maintained under a 12:12 h light/dark cycle, with 10–15 air changes per hour, relative humidity of 40–60%, and room temperature 23–24 °C. Prior to the start of the study, all animals were monitored twice daily during routine animal health rounds. On day 0, mice were anesthetized by intraperitoneal (IP) injection of Ketamine (100 mg/kg) and Xylazine (10 mg/kg), their backs were shaved, the surgery area was sterilized with iodine and 70% alcohol, and a 6 mm, full-thickness wound was created on their dorsal side using a sterile biopsy punch. Each wound was inoculated with bioluminescent *P. aeruginosa* strain PAO1::*lux* in 25 µL of PBS (approximately 5 × 10^7^ CFU in the PAM1 experiment and 1 × 10^7^ CFU in the PAM2 experiment) and covered with a Tegaderm^TM^ bandage (3M, Maplewood, MN, USA). Mice were single-housed from day 0 (inoculation) through day 15 and then returned to group housing. The analgesic Buprenorphine was given to mice intramuscularly at 0.05 mg/kg once on day 0, immediately following surgery, when the animals were still sedated. Additional Buprenorphine was administered to mice demonstrating clinical signs indicative of pain and distress. There were three treatment groups of five mice each in the PAM1 experiment (phage treatment, PBS—negative control, and ceftazidime—positive control). There were two treatment groups in the PAM2 experiment (phage treatment, 20 mice, and PBS treatment, 15 mice). For phage treatment, mice received 1 × 10^9^ PFU of a cocktail (2 × 10^8^ PFU of each phage in the cocktail) in 25 µL of PBS topically under the Tegaderm dressing and the same dose in 200 µL of PBS IP, once a day. Mice not receiving phage treatment instead received doses of PBS topically and IP once a day or ceftazidime IP, in a dose of 410 mg/kg, at 5 µL/g, twice a day. Treatments were administered at 4 h (day 0), and then once daily on days 1–4 (PAM1 experiment) or 1–3 (PAM2 experiment), for a total of five or four treatments, respectively. On day 7 post-infection, Tegaderm^TM^ dressings were removed. To assess the efficacy of treatments, multiple parameters were measured, including survival, body weight, clinical scores, wound size and healing, and bioluminescent signal in the wound. Mouse weights and clinical scores were monitored and recorded daily. An in vivo imaging system (IVIS; PerkinElmer, Waltham, MA, USA) was used to measure the bioluminescent signal of PAO1::*lux* as a means to visualize and perform relative quantification of bacterial burden in the wound beds over the course of the PAM1 experiment. Wound size was measured and compared for all mice in all treatment groups using a Silhouette wound measurement device (Aranz Medical Ltd., Christchurch, New Zealand) on days 0, 6, 8, 10, 13, and 15 post-infection (PAM1 experiment) and days 0, 6, 10, and 15 (PAM2 experiment). Mice determined to have reached the experimental endpoint were humanely euthanized. Survival data were recorded and presented as survival graphs. Statistical analyses were completed using a Gehan–Breslow–Wilcoxon test (for survival) and Mann–Whitney *t*-test (for wound healing). Significance was established at *p* < 0.05.

## Figures and Tables

**Table 1:** Characteristics of 25 *P. aeruginosa* phages used in this work.

Phage ID	Genome Size, bp	Family *	Genus	Host Range	Anti-Biofilm Effect		Phage Receptor
					Dispersal	Killing	
EPa2	43,299	Unclassified	*Bruynoghevirus*	32.7%	+	+	T4P, A band (1)
EPa4	45,439	Unclassified	*Bruynoghevirus*	31.4%	+	+	T4P, A band (1)
EPa5	64,096	*Mesyanzhinovviridae*	*Epaquintavirus*	18.6%	–	+	T4P, LPS core (2)
EPa6	66,031	*Lindbergviridae*	*Pbunavirus*	46.2%	–	+	T4P, LPS core (2)
EPa7	65,629	*Lindbergviridae*	*Pbunavirus*	44.2%	–	+	T4P, LPS core (2)
EPa10	66,774	*Lindbergviridae*	*Pbunavirus*	40.4%	–	+	T4P, L-B, VL-B (3)
EPa11	66,800	*Lindbergviridae*	*Pbunavirus*	51.3%	–	+	T4P, L-B, VL-B (3)
EPa12	66,520	*Lindbergviridae*	*Pbunavirus*	46.2%	–	+	T4P, L-B, VL-B (3)
EPa13	65,680	*Lindbergviridae*	*Pbunavirus*	42.3%	–	+	T4P, A, B, L-B, VL-B (4)
EPa14	65,797	*Lindbergviridae*	*Pbunavirus*	42.3%	–	+	T4P, A, B, L-B, VL-B (4)
EPa15	66,002	*Lindbergviridae*	*Pbunavirus*	54.5%	–	+	T4P, L-B, VL-B (3)
EPa16	88,727	*Vandenendeviridae*	*Nankokuvirus*	35.9%	–	+	Unknown **
EPa17	88,859	*Vandenendeviridae*	*Nankokuvirus*	30.8%	–	+	Unknown **
EPa18	88,109	*Vandenendeviridae*	*Nankokuvirus*	37.8%	–	+	Unknown **
EPa20	66,505	*Lindbergviridae*	*Pbunavirus*	42.3%	–	+	T4P, L-B, VL-B (3)
EPa21	66,764	*Lindbergviridae*	*Pbunavirus*	39.7%	–	+	T4P, L-B, VL-B (3)
EPa22	65,897	*Lindbergviridae*	*Pbunavirus*	51.9%	–	+	T4P, A, B, L-B, VL-B (4)
EPa24	88,728	*Vandenendeviridae*	*Nankokuvirus*	46.8%	–	+	T4P, LPS core (2)
EPa25	66,811	*Lindbergviridae*	*Pbunavirus*	28.2%	–	+	T4P, L-B, VL-B (3)
EPa26	88,805	*Vandenendeviridae*	*Nankokuvirus*	42.9%	–	+	T4P, LPS core (2)
EPa33	64,021	Unclassified	*Hollowayvirus*	30.1%	–	+	T4P, A, B (5)
EPa38	61,775	*Mesyanzhinovviridae*	*Yuavirus*	7.7%	–	+	T4P, LPS core (2)
EPa39	66,708	*Lindbergviridae*	*Pbunavirus*	45.5%	–	+	T4P, L-B, VL-B (3)
EPa40	42,788	*Unclassified*	*Septimatrevirus*	32.7%	–	+	T4P, A band (1)
EPa43	64,323	*Mesyanzhinoviridae*	*Epaquintavirus*	16.7%	–	+	T4P, LPS core (2)

Notes: * Phage taxonomy at the family level is in the process of updating by the International Committee on Taxonomy of Viruses (ICTV). Until recently, genera *Bruynoghevirus* and *Hollowayvirus* belonged to the family *Podoviridae*, and genus *Septimatrevirus* belonged to the family *Siphoviridae* formerly established based on virion morphology; now these phages are not classified at the family level. T4P, type IV pili; A band, common polysaccharide antigen (CPA) chain of lipopolysaccharide (LPS); B band, O-specific antigen (OSA) polysaccharide chain of the LPS; L-B, long B band; VL-B, very long B band. ** These phages are not active against *P. aeruginosa* strain PAO1 and its derivatives.

**Table 2:** Predicted complementary host ranges of *P. aeruginosa* phages for pairwise mixes.

Phage	Host Range (%)	Phage	Host Range (%)	Phage	Host Range (%)
EPa11	51.3	EPa11 + EPa24	59.0	EPa17 + EPa40	48.7
EPa16	35.9	EPa11 + EPa40	53.8	EPa17 + EPa43	38.5
EPa17	30.8	EPa11 + EPa43	55.8	EPa22 + EPa24	65.4
EPa22	51.9	EPa16 + EPa17	51.3	EPa22 + EPa40	57.1
EPa24	46.8	EPa16 + EPa22	60.9	EPa22 + EPa43	54.5
EPa40	32.7	EPa16 + EPa24	51.3	EPa24 + EPa40	50.0
EPa43	16.7	EPa16 + EPa40	44.2	EPa24 + EPa43	52.6
EPa11 + EPa16	57.1	EPa16 + EPa43	42.3	EPa40 + EPa43	39.1
EPa11 + EPa17	64.1	EPa17 + EPa22	61.6	Mix-7 *	78.2
EPa11 + EPa22	62.2	EPa17 + EPa24	60.1	Mix-5 **	76.3

Notes: * Mix-7: EPa11, EPa16, EPa17, EPa22, EPa24, EPa40, and EPa43. ** Mix-5: EPa11, EPa17, EPa22, EPa24, and EPa43.

**Table 3:** Frequencies of *P. aeruginosa* resistance to single phages and their pairwise mixes.

Phage	Resistance Frequency		Phage	Resistance Frequency	
	To Single	To Mix		To Single	To Mix
EPa11	7.54 × 10^−6^	5.70 × 10^−6^	EPa11	7.54 × 10^−6^	2.63 × 10^−8^
EPa16	1.33 × 10^−6^		EPa17	2.19 × 10^−7^	
EPa11	7.54 × 10^−6^	6.40 × 10^−6^	EPa11	7.54 × 10^−6^	8.07 × 10^−6^
EPa22	3.42 × 10^−6^		EPa24	9.39 × 10^−6^	
EPa11	7.54 × 10^−6^	1.58 × 10^−6^	EPa11	7.54 × 10^−6^	<8.77 × 10^−9^
EPa40	2.53 × 10^−7^		EPa43	<8.77 × 10^−9^	
EPa16	1.33 × 10^−6^	1.67 × 10^−7^	EPa16	1.33 × 10^−6^	2.98 × 10^−6^
EPa17	2.19 × 10^−7^		EPa22	3.42 × 10^−6^	
EPa16	1.33 × 10^−6^	5.35 × 10^−6^	EPa16	1.33 × 10^−6^	1.01 × 10^−6^
EPa24	9.39 × 10^−6^		EPa40	2.53 × 10^−7^	
EPa16	1.33 × 10^−6^	<8.77 × 10^−9^	EPa17	2.19 × 10^−7^	4.39 × 10^−8^
EPa43	<8.77 × 10^−9^		EPa22	3.42 × 10^−6^	
EPa17	2.19 × 10^−7^	3.51 × 10^−8^	EPa17	2.19 × 10^−7^	8.77 × 10^−8^
EPa24	9.39 × 10^−6^		EPa40	2.53 × 10^−7^	
EPa17	2.19 × 10^−7^	<8.77 × 10^−9^	EPa22	3.42 × 10^−6^	6.93 × 10^−6^
EPa43	<8.77 × 10^−9^		EPa24	9.39 × 10^−6^	
EPa22	3.42 × 10^−6^	1.19 × 10^−5^	EPa22	3.42 × 10^−6^	<8.77 × 10^−9^
EPa40	2.53 × 10^−7^		EPa43	<8.77 × 10^−9^	
EPa24	9.39 × 10^−6^	1.19 × 10^−5^	EPa24	9.39 × 10^−6^	<8.77 × 10^−9^
EPa40	2.53 × 10^−7^		EPa43	<8.77 × 10^−9^	
EPa40	2.53 × 10^−7^	<8.77 × 10^−9^	11 + 16 + 17 + 22 + 24 + 40 + 43		<8.77 × 10^−9^
EPa43	<8.77 × 10^−9^				

**Table 4:** Phage cocktails PAM1 and PAM2.

Cocktail	Phage	Family	Genus	Host Range (%)	Host Receptor	Cocktail Activity (%)
PAM1	EPa2	Unclassified	*Bruynoghevirus*	32.7	T4P, A band (1)	55.8
	EPa4	Unclassified	*Bruynoghevirus*	31.4	T4P, A band (1)	
	EPa5	*Mesyanzhinovviridae*	*Epaquintavirus*	18.6	T4P, LPS core (2)	
	EPa6	*Lindbergviridae*	*Pbunavirus*	46.2	T4P, LPS core (2)	
	EPa17	*Vandenendeviridae*	*Nankokuvirus*	30.8	Unknown	
PAM2	EPa11	*Lindbergviridae*	*Pbunavirus*	51.3	T4P, L-B, VL-B (3)	76.3
	EPa17	*Vandenendeviridae*	*Nankokuvirus*	30.8	Unknown	
	EPa22	*Lindbergviridae*	*Pbunavirus*	51.9	T4P, A, B, L-B, VL-B (4)	
	EPa24	*Vandenendeviridae*	*Nankokuvirus*	46.8	T4P, LPS core (2)	
	EPa43	*Mesyanzhinovviridae*	*Epaquintavirus*	14.1	T4P, LPS core (2)	

**Table 5:** Twelve knockout mutants of *P. aeruginosa* PAO1 used for phage receptor identification.

Mutant	Phenotype	Source	Mutant	Phenotype	Source
*rmd*	No A band LPS	Dr. Rob Lavigne (Katholieke Universiteit Leuven, Leuven, Belgium)	*wzz1*	No long B band	Dr. Karen Maxwell (University of Toronto, Toronto, Canada)
*wbpL*	A/B band synthesis initiation defect		*wzz2*	No very long B band	
*rmlC* *	Truncated LPS core, no A, B bands		*wzz1,2*	No long/very long B band	
*fliA*	No flagella		*wapQ*	No phosphate in LPS inner core	
*pilA*	No type IV pili		*wbpM*	No B band	
*fliA algC pilA*	No flagella, A band, type IV pili		*rmlC* *	Truncated LPS core, no A, B bands	

* Two *rmlC* mutants acquired from two different sources were tested.

## Data Availability

The original contributions presented in this study are included in the article. Further inquiries can be directed to the corresponding author.

## Supplementary Materials

The following supporting information can be downloaded at: https://www.mdpi.com/article/10.3390/antibiotics15010075/s1, Table S1: Phage plaque assay results; Table S2: *P. aeruginosa* strains used in this work; Table S3: Identification of phage receptors by plating on *P. aeruginosa* PAO1 mutants; Figure S1: Stability of single *P. aeruginosa* phages (a) and their pairwise mixes (b) for nine months at 4 °C.

## Abbreviations

The following abbreviations are used in this manuscript:

CAZ	Ceftazidime
CFU	Colony-forming unit
EOP	Efficiency of plating
HIB	Heart Infusion Broth
PAM	*Pseudomonas aeruginosa* phage mix
LPS	Lipopolysaccharide
MDR	Multidrug-resistant
MLST	Multilocus sequence typing
MOI	Multiplicity of infection
MRSN	Multidrug-Resistant Organism Repository and Surveillance Network
PDR	Pandrug-resistant
PFU	Plaque-forming unit
ST	Sequence type
T4P	Type IV pili
WRAIR	Walter Reed Army Institute of Research
XDR	Extensively drug-resistant

## References

[1] Reynolds D., Kollef M. (2021). The epidemiology and pathogenesis and treatment of *Pseudomonas aeruginosa* infections: An update. Drugs.

[2] Wood S.J., Kuzel T.M., Shafikhani S.H. (2023). *Pseudomonas aeruginosa*: Infections, animal modeling, and therapeutics. Cells.

[3] Keen E.F., Murray C.K., Robinson B.J., Hospenthal D.R., Co E.-M.A., Aldous W.K. (2010). Changes in the incidences of multidrug-resistant and extensively drug-resistant organisms isolated in a military medical center. Infect. Control Hosp. Epidemiol..

[4] Azzopardi E.A., Azzopardi E., Camilleri L., Villapalos J., Boyce D.E., Dziewulski P., Dickson W.A., Whitaker I.S. (2014). Gram negative wound infection in hospitalised adult burn patients—Systematic review and metanalysis. PLoS ONE.

[5] Norbury W., Herndon D.N., Tanksley J., Jeschke M.G., Finnerty C.C. (2016). Infection in burns. Surg. Infect..

[6] Maslova E., Eisaiankhongi L., Sjöberg F., McCarthy R.R. (2021). Burns and biofilms: Priority pathogens and in vivo models. NPJ Biofilms Microbiomes.

[7] Ghasemian S., Karami-Zarandi M., Heidari H., Khoshnood S., Kouhsari E., Ghafourian S., Maleki A., Kazemian H. (2023). Molecular characterizations of antibiotic resistance, biofilm formation, and virulence determinants of *Pseudomonas aeruginosa* isolated from burn wound infection. J. Clin. Lab. Anal..

[8] Burns T.C., Stinner D.J., Mack A.W., Potter B.K., Beer R., Eckel T.T., Possley D.R., Beltran M.J., Hayda R.A., Andersen R.C. (2012). Microbiology and injury characteristics in severe open tibia fractures from combat. J. Trauma Acute Care Surg..

[9] Akers K.S., Mende K., Cheatle K.A., Zera W.C., Yu X., Beckius M.L., Aggarwal D., Li P., Sanchez C.J., Wenke J.C. (2014). Biofilms and persistent wound infections in United States military trauma patients: A case-control analysis. BMC Infect. Dis..

[10] Tribble D.R., Krauss M.R., Murray C.K., Warkentien T.E., Lloyd B.A., Ganesan A., Greenberg L., Xu J., Li P., Carson M.L. (2018). Epidemiology of trauma-related infections among a combat casualty cohort after initial hospitalization: The trauma infectious disease outcomes study. Surg. Infect..

[11] Mende K., Akers K.S., Tyner S.D., Bennett J.W., Simons M.P., Blyth D.M., Li P., Stewart L., Tribble D.R. (2022). Multidrug-resistant and virulent organisms trauma infections: Trauma Infectious Disease Outcomes Study Initiative. Mil. Med..

[12] Serra R., Grande R., Butrico L., Rossi A., Settimio U.F., Caroleo B., Amato B., Gallelli L., de Franciscis S. (2015). Chronic wound infections: The role of *Pseudomonas aeruginosa* and *Staphylococcus aureus*. Expert Rev. Anti-Infect. Ther..

[13] Ruffin M., Brochiero E. (2019). Repair process impairment by *Pseudomonas aeruginosa* in epithelial tissues: Major features and potential therapeutic avenues. Front. Cell. Infect. Microbiol..

[14] Sachdeva C., Satyamoorthy K., Murali T.S. (2022). Microbial interplay in skin and chronic wounds. Curr. Clin. Microbiol. Rep..

[15] Phan S., Feng C.H., Huang R., Lee Z.X., Moua Y., Phung O.J., Lenhard J.R. (2023). Relative abundance and detection of *Pseudomonas aeruginosa* from chronic wound infections globally. Microorganisms.

[16] Langendonk R.F., Neill D.R., Fothergill J.L. (2021). The building blocks of antimicrobial resistance in *Pseudomonas aeruginosa*: Implications for current resistance-breaking therapies. Front. Cell. Infect. Microbiol..

[17] Ciofu O., Tolker-Nielsen T. (2019). Tolerance and resistance of *Pseudomonas aeruginosa* biofilms to antimicrobial agents—How *P*. aeruginosa can escape antibiotics. Front. Microbiol..

[18] Thi M.T.T., Wibowo D., Rehm B.H.A. (2020). *Pseudomonas aeruginosa* biofilms. Int. J. Mol. Sci..

[19] Kamal S., Varshney K., Uayan D.J., Tenorio B.G., Pillay P., Sava S.T. (2024). Risk factors and clinical characteristics of pandrug-resistant *Pseudomonas aeruginosa*. Cureus.

[20] Krylov V.N. (2014). Bacteriophages of *Pseudomonas aeruginosa*: Long-term prospects for use in phage therapy. Adv. Virus Res..

[21] Nikolich M.P., Filippov A.A. (2020). Bacteriophage therapy: Developments and directions. Antibiotics.

[22] Holger D., Kebriaei R., Morrisette T., Lev K., Alexander J., Rybak M. (2021). Clinical pharmacology of bacteriophage therapy: A focus on multidrug-resistant *Pseudomonas aeruginosa* infections. Antibiotics.

[23] Strathdee S.A., Hatfull G.F., Mutalik V.K., Schooley R.T. (2023). Phage therapy: From biological mechanisms to future directions. Cell.

[24] Cui L., Watanabe S., Miyanaga K., Kiga K., Sasahara T., Aiba Y., Tan X.E., Veeranarayanan S., Thitiananpakorn K., Nguyen H.M. (2024). A comprehensive review on phage therapy and phage-based drug development. Antibiotics.

[25] Alipour-Khezri E., Skurnik M., Zarrini G. (2024). *Pseudomonas aeruginosa* bacteriophages and their clinical applications. Viruses.

[26] Alves D.R., Perez-Esteban P., Kot W., Bean J.E., Arnot T., Hansen L.H., Enright M.C., Jenkins A.T.A. (2016). A novel bacteriophage cocktail reduces and disperses *Pseudomonas aeruginosa* biofilms under static and flow conditions. Microb. Biotechnol..

[27] Chegini Z., Khoshbayan A., Moghadam M.T., Farahani I., Jazireian P., Shariati A. (2020). Bacteriophage therapy against *Pseudomonas aeruginosa* biofilms: A review. Ann. Clin. Microbiol. Antimicrob..

[28] Amankwah S., Adisu M., Gorems K., Abdella K., Kassa T. (2022). Assessment of phage-mediated inhibition and removal of multidrug-resistant *Pseudomonas aeruginosa* biofilm on medical implants. Infect. Drug Resist..

[29] Saussereau E., Debarbieux L. (2012). Bacteriophages in the experimental treatment of *Pseudomonas aeruginosa* infections in mice. Adv. Virus Res..

[30] Soothill J. (2013). Use of bacteriophages in the treatment of *Pseudomonas aeruginosa* infections. Expert Rev. Anti Infect. Ther..

[31] Pires D.P., Vilas Boas D., Sillankorva S., Azeredo J. (2015). Phage therapy: A step forward in the treatment of *Pseudomonas aeruginosa* infections. J. Virol..

[32] Melo L.D.R., Oliveira H., Pires D.P., Dabrowska K., Azeredo J. (2020). Phage therapy efficacy: A review of the last 10 years of preclinical studies. Crit. Rev. Microbiol..

[33] Khan F.M., Manohar P., Gondil V.S., Mehra N., Oyejobi G.K., Odiwuor N., Ahmad T., Huang G. (2023). The applications of animal models in phage therapy: An update. Hum. Vaccines Immunother..

[34] Basu S., Agarwal M., Kumar Bhartiya S., Nath G., Kumar Shukla V. (2015). An in vivo wound model utilizing bacteriophage therapy of *Pseudomonas aeruginosa* biofilms. Ostomy Wound Manag..

[35] Engeman E., Freyberger H.R., Corey B.W., Ward A.M., He Y., Nikolich M.P., Filippov A.A., Tyner S.D., Jacobs A.C. (2021). Synergistic killing and re-sensitization of *Pseudomonas aeruginosa* to antibiotics by phage-antibiotic combination treatment. Pharmaceuticals.

[36] McVay C.S., Velásquez M., Fralick J.A. (2007). Phage therapy of *Pseudomonas aeruginosa* infection in a mouse burn wound model. Antimicrob. Agents Chemother..

[37] Kumari S., Harjai K., Chhibber S. (2009). Bacteriophage treatment of burn wound infection caused by *Pseudomonas aeruginosa* PAO in BALB/c mice. Am. J. Biomed. Sci..

[38] Holguín A.V., Rangel G., Clavijo V., Prada C., Mantilla M., Gomez M.C., Kutter E., Taylor C., Fineran P.C., González Barrios A.F. (2015). Phage ΦPan70, a putative temperate phage, controls *Pseudomonas aeruginosa* in planktonic, biofilm and burn mouse model assays. Viruses.

[39] Kamer A.M.A., Abdelaziz A.A., Nosair A.M., Al-Madboly L.A. (2022). Characterization of newly isolated bacteriophage to control multi-drug resistant *Pseudomonas aeruginosa* colonizing incision wounds in a rat model: In vitro and in vivo approach. Life Sci..

[40] Wang J., Meng W., Zhang K., Wang J., Lu B., Wang R., Jia K. (2022). Topically applied bacteriophage to control multi-drug resistant *Pseudomonas aeruginosa*-infected wounds in a New Zealand rabbit model. Front. Microbiol..

[41] Ghanaim A.M., Foaad M.A., Gomaa E.Z., El Dougdoug K.A., Mohamed G.E., Arisha A.H., Khamis T. (2023). Bacteriophage therapy as an alternative technique for treatment of multidrug-resistant bacteria causing diabetic foot infection. Int. Microbiol..

[42] Jennes S., Merabishvili M., Soentjens P., Pang K.W., Rose T., Keersebilck E., Soete O., François P.-M., Teodorescu S., Verween G. (2017). Use of bacteriophages in the treatment of colistin-only-sensitive *Pseudomonas aeruginosa* septicaemia in a patient with acute kidney injury—A case report. Crit. Care.

[43] Chan B.K., Turner P.E., Kim S., Mojibian H.R., Elefteriades J.A., Narayan D. (2018). Phage treatment of an aortic graft infected with *Pseudomonas aeruginosa*. Evol. Med. Public Health.

[44] Aslam S., Lampley E., Wooten D., Karris M., Benson C., Strathdee S., Schooley R.T. (2020). Lessons learned from the first 10 consecutive cases of intravenous bacteriophage therapy to treat multidrug-resistant bacterial infections at a single center in the United States. Open Forum Infect. Dis..

[45] Otava U.E., Tervo L., Havela R., Vuotari L., Ylänne M., Asplund A., Patpatia S., Kiljunen S. (2024). Phage-antibiotic combination therapy against recurrent *Pseudomonas* septicaemia in a patient with an arterial stent. Antibiotics.

[46] Green S.I., Clark J.R., Santos H.H., Weesner K.E., Salazar K.C., Aslam S., Campbell J.W., Doernberg S.B., Blodget E., Morris M.J. (2023). A retrospective, observational study of 12 cases of expanded-access customized phage therapy: Production, characteristics, and clinical outcomes. Clin. Infect. Dis..

[47] Onallah H., Hazan R., Nir-Paz R., Brownstein M.J., Fackler J.R., Horne B., Hopkins R., Basu S., Yerushalmy O., Alkalay-Oren S. (2023). Refractory *Pseudomonas aeruginosa* infections treated with phage PASA16: A compassionate use case series. Med.

[48] Wright A., Hawkins C.H., Anggård E.E., Harper D.R. (2009). A controlled clinical trial of a therapeutic bacteriophage preparation in chronic otitis due to antibiotic-resistant *Pseudomonas aeruginosa*; a preliminary report of efficacy. Clin. Otolaryngol..

[49] Jault P., Leclerc T., Jennes S., Pirnay J.P., Que Y.A., Resch G., Rousseau A.F., Ravat F., Carsin H., Le Floch R. (2019). Efficacy and tolerability of a cocktail of bacteriophages to treat burn wounds infected by *Pseudomonas aeruginosa* (PhagoBurn): A randomised, controlled, double-blind phase 1/2 trial. Lancet Infect. Dis..

[50] Yang Q., Le S., Zhu T., Wu N. (2023). Regulations of phage therapy across the world. Front. Microbiol..

[51] Ceyssens P.-J., Lavigne R., Mattheus W., Chibeu A., Hertveldt K., Mast J., Robben J., Volckaert G. (2006). Genomic analysis of *Pseudomonas aeruginosa* phages LKD16 and LKA1: Establishment of the ɸKMV subgroup within the T7 supergroup. J. Bacteriol..

[52] Ceyssens P.-J., Hertveldt K., Ackermann H.-W., Noben J.-P., Demeke M., Volckaert G., Lavigne R. (2008). The intron-containing genome of the lytic *Pseudomonas* phage LUZ24 resembles the temperate phage PaP3. Virology.

[53] Debarbieux L., Leduc D., Maura D., Morello E., Criscuolo A., Grossi O., Balloy V., Touqui L. (2010). Bacteriophages can treat and prevent *Pseudomonas aeruginosa* lung infections. J. Infect. Dis..

[54] Garbe J., Bunk B., Rohde M., Schobert M. (2011). Sequencing and characterization of *Pseudomonas aeruginosa* phage JG004. BMC Microbiol..

[55] Essoh C., Blouin Y., Loukou G., Cablanmian A., Lathro S., Kutter E., Thien H.V., Vergnaud G., Pourcel C. (2013). The susceptibility of *Pseudomonas aeruginosa* strains from cystic fibrosis patients to bacteriophages. PLoS ONE.

[56] Kwiatek M., Mizak L., Parasion S., Gryko R., Olender A., Niemcewicz M. (2015). Characterization of five newly isolated bacteriophages active against *Pseudomonas aeruginosa* clinical strains. Folia Microbiol..

[57] Tang C., Deng C., Zhang Y., Xiao C., Wang J., Rao X., Hu F., Lu S. (2018). Characterization and genomic analyses of *Pseudomonas aeruginosa* podovirus TC6: Establishment of genus *Pa11virus*. Front. Microbiol..

[58] Olszak T., Danis-Wlodarczyk K., Arabski M., Gula G., Maciejewska B., Wasik S., Lood C., Higgins G., Harvey B.J., Lavigne R. (2019). *Pseudomonas aeruginosa* PA5oct jumbo phage impacts planktonic and biofilm population and reduces its host virulence. Viruses.

[59] Knezevic P., Kostanjsek R., Obreht D., Petrovic O. (2009). Isolation of *Pseudomonas aeruginosa* specific phages with broad activity spectra. Curr. Microbiol..

[60] Garbe J., Wesche A., Bunk B., Kazmierczak M., Selezska K., Rohde C., Sikorski J., Rohde M., Jahn D., Schobert M. (2010). Characterization of JG024, a *Pseudomonas aeruginosa* PB1-like broad host range phage under simulated infection conditions. BMC Microbiol..

[61] Essoh C., Latino L., Midoux C., Blouin Y., Loukou G., Nguetta S.-P.A., Lathro S., Cablanmian A., Kouassi A.K., Vergnaud G. (2015). Investigation of a large collection of *Pseudomonas aeruginosa* bacteriophages collected from a single environmental source in Abidjan, Côte d’Ivoire. PLoS ONE.

[62] Mattila S., Ruotsalainen P., Jalasvuori M. (2015). On-demand isolation of bacteriophages against drug-resistant bacteria for personalized phage therapy. Front. Microbiol..

[63] Latz S., Krüttgen A., Häfner H., Buhl E.M., Ritter K., Horz H.-P. (2017). Differential effect of newly isolated phages belonging to PB1-like, ɸKZ-like and LUZ24-like viruses against multi-drug resistant *Pseudomonas aeruginosa* under varying growth conditions. Viruses.

[64] Karumidze N., Thomas J.A., Kvatadze N., Goderdzishvili M., Hakala K.W., Weintraub S.T., Alavidze Z., Hardies S.C. (2012). Characterization of lytic *Pseudomonas aeruginosa* bacteriophages via biological properties and genomic sequences. Appl. Microbiol. Biotechnol..

[65] Danis-Wlodarczyk K., Olszak T., Arabski M., Wasik S., Majkowska-Skrobek G., Augustyniak D., Gula G., Briers Y., Jang H.B., Vandenheuvel D. (2015). Characterization of the newly isolated lytic bacteriophages KTN6 and KT28 and their efficacy against *Pseudomonas aeruginosa* biofilm. PLoS ONE.

[66] Forti F., Roach D.R., Cafora M., Pasini M.E., Horner D.S., Fiscarelli E.V., Rossitto M., Cariani L., Briani F., Debarbieux L. (2018). Design of a broad-range bacteriophage cocktail that reduces *Pseudomonas aeruginosa* biofilms and treats acute infections in two animal models. Antimicrob. Agents Chemother..

[67] Hall A.R., De Vos D., Friman V.P., Pirnay J.-P., Buckling A. (2012). Effects of sequential and simultaneous applications of bacteriophages on populations of *Pseudomonas aeruginosa* in vitro and in wax moth larvae. Appl. Environ. Microbiol..

[68] Feary T.W., Fisher E., Fisher T.N. (1963). Lysogeny and phage resistance in *Pseudomonas aeruginosa*. Proc. Soc. Exp. Biol. Med..

[69] Le S., Yao X., Lu S., Tan Y., Rao X., Li M., Jin X., Wang J., Zhao Y., Wu N.C. (2014). Chromosomal DNA deletion confers phage resistance to *Pseudomonas aeruginosa*. Sci. Rep..

[70] Friman V.P., Soanes-Brown D., Sierocinski P., Molin S., Johansen H.K., Merabishvili M., Pirnay J.-P., De Vos D., Buckling A. (2016). Pre-adapting parasitic phages to a pathogen leads to increased pathogen clearance and lowered resistance evolution with *Pseudomonas aeruginosa* cystic fibrosis bacterial isolates. J. Evol. Biol..

[71] Pinto A.M., Faustino A., Pastrana L.M., Bañobre-López M., Sillankorva S. (2021). *Pseudomonas aeruginosa* PAO1 in vitro time-kill kinetics using single phages and phage formulations—Modulating death, adaptation, and resistance. Antibiotics.

[72] Wright R.C.T., Friman V.-P., Smith M.C.M., Brockhurst M.A. (2019). Resistance evolution against phage combinations depends on the timing and order of exposure. mBio.

[73] Boon M., Holtappels D., Lood C., van Noort V., Lavigne R. (2020). Host range expansion of *Pseudomonas* virus LUZ7 is driven by a conserved tail fiber mutation. Phage.

[74] Vaitekenas A., Tai A.S., Ramsay J.P., Stick S.M., Kicic A. (2021). *Pseudomonas aeruginosa* resistance to bacteriophages and its prevention by strategic therapeutic cocktail formulation. Antibiotics.

[75] Fu W., Forster T., Mayer O., Curtin J.J., Lehman S.M., Donlan R.M. (2010). Bacteriophage cocktail for the prevention of biofilm formation by *Pseudomonas aeruginosa* on catheters in an in vitro model system. Antimicrob. Agents Chemother..

[76] Chan B.K., Abedon S.T., Loc-Carrillo C. (2013). Phage cocktails and the future of phage therapy. Future Microbiol..

[77] Merabishvili M., Pirnay J.-P., Vos D.D. (2018). Guidelines to compose an ideal bacteriophage cocktail. Methods Mol. Biol..

[78] Kovacs C.J., Rapp E.M., Rankin W.R., McKenzie S.M., Brasko B.K., Hebert K.E., Bachert B.A., Kick A.R., Burpo F.J., Barnhill J.C. (2024). Combinations of bacteriophage are efficacious against multidrug-resistant *Pseudomonas aeruginosa* and enhance sensitivity to carbapenem antibiotics. Viruses.

[79] Marchi J., Minh C.N.N., Debarbieux L., Weitz J.S. (2025). Multi-strain phage induced clearance of bacterial infections. PLoS Comput. Biol..

[80] Farlow J., Freyberger H.R., He Y., Ward A.M., Rutvisuttinunt W., Li T., Campbell R., Jacobs A.C., Nikolich M.P., Filippov A.A. (2020). Complete genome sequences of 10 phages lytic against multidrug-resistant *Pseudomonas aeruginosa*. Microbiol. Resour. Announc..

[81] Campbell R.A., Farlow J., Freyberger H.R., He Y., Ward A.M., Ellison D.W., Getnet D., Swierczewski B.E., Nikolich M.P., Filippov A.A. (2021). Genome sequences of 17 diverse *Pseudomonas aeruginosa* phages. Microbiol. Resour. Announc..

[82] Ondov B.D., Treangen T.J., Melsted P., Mallonee A.B., Bergman N.H., Koren S., Phillippy A.M. (2016). Mash: Fast genome and metagenome distance estimation using MinHash. Genome Biol..

[83] Cook R., Brown N., Redgwell T., Rihtman B., Barnes M., Clokie M., Stekel D.J., Hobman J., Jones M.A., Millard A. (2021). INfrastructure for a PHAge REference Database: Identification of large-scale biases in the current collection of cultured phage genomes. Phage.

[84] Meier-Kolthoff J.P., Göker M. (2017). VICTOR: Genome-based phylogeny and classification of prokaryotic viruses. Bioinformatics.

[85] Letunic I., Bork P. (2024). Interactive Tree of Life (iTOL) v6: Recent updates to the phylogenetic tree display and annotation tool. Nucleic Acids Res..

[86] Hockenberry A.J., Wilke C.O. (2021). BACPHLIP: Predicting bacteriophage lifestyle from conserved protein domains. PeerJ.

[87] Byrne M., Kropinski A.M. (2005). The genome of the *Pseudomonas aeruginosa* generalized transducing bacteriophage F116. Gene.

[88] Alcock B.P., Raphenya A.R., Lau T.T.Y., Tsang K.K., Bouchard M., Edalatmand A., Huynh W., Nguyen A.-L.V., Cheng A.A., Liu S. (2019). CARD 2020: Antibiotic resistome surveillance with the comprehensive antibiotic resistance database. Nucleic Acids Res..

[89] Chen L. (2004). VFDB: A reference database for bacterial virulence factors. Nucleic Acids Res..

[90] Lebreton F., Snesrud E., Hall L., Mills E., Galac M., Stam J., Ong A., Maybank R., Kwak Y.I., Johnson S. (2021). A panel of diverse *Pseudomonas aeruginosa* clinical isolates for research and development. JAC Antimicrob. Resist..

[91] Treepong P., Kos V.N., Guyeux C., Blanc D.S., Bertrand X., Valot B., Hocquet D. (2018). Global emergence of the widespread *Pseudomonas aeruginosa* ST235 clone. Clin. Microbiol. Infect..

[92] Horcajada J.P., Montero M., Oliver A., Sorlí L., Luque S., Gómez-Zorrilla S., Benito N., Grau S. (2019). Epidemiology and treatment of multidrug-resistant and extensively drug-resistant *Pseudomonas aeruginosa* infections. Clin. Microbiol. Rev..

[93] Del Barrio-Tofiño E., López-Causapé C., Oliver A. (2020). *Pseudomonas aeruginosa* epidemic high-risk clones and their association with horizontally-acquired β-lactamases: 2020 update. Int. J. Antimicrob. Agents.

[94] Stribling W., Hall L.R., Powell A., Harless C., Martin M.J., Corey B.W., Snesrud E., Ong A., Maybank R., Stam J. (2024). Detecting, mapping, and suppressing the spread of a decade-long *Pseudomonas aeruginosa* nosocomial outbreak with genomics. eLife.

[95] Hughes K.A., Sutherland I.W., Jones M.V. (1998). Biofilm susceptibility to bacteriophage attack: The role of phage-borne polysaccharide depolymerase. Microbiology.

[96] Kim H.-J., Kim J.-H., Son J.H., Seo H.-J., Park S.-J., Paek N.-S., Kim S.-K. (2004). Characterization of bacteriocin produced by *Lactobacillus bulgaricus*. J. Microbiol. Biotechnol..

[97] Liao C., Liu X., Shan L. (2014). Optimization of liquid media and biosafety assessment for algae-lysing bacterium NP23. Can. J. Microbiol..

[98] Othman M., Ariff A.B., Wasoh H., Kapri M.R., Halim M. (2017). Strategies for improving production performance of probiotic *Pediococcus acidilactici* viable cell by overcoming lactic acid inhibition. AMB Express.

[99] Boukhris I., Smaoui S., Ennouri K., Morjene N., Farhat-Khemakhem A., Blibech M., Alghamdi O.A., Chouayekh H. (2020). Towards understanding the antagonistic activity of phytic acid against common foodborne bacterial pathogens using a general linear model. PLoS ONE.

[100] Tang H.W., Abbasiliasi S., Murugan P., Tam Y.J., Ng H.S., Tan J.S. (2020). Influence of freeze-drying and spray-drying preservation methods on survivability rate of different types of protectants encapsulated *Lactobacillus acidophilus* FTDC 3081. Biosci. Biotechnol. Biochem..

[101] Purnomo A.S., Sariwati A., Kamei I. (2020). Synergistic interaction of a consortium of the brown-rot fungus *Fomitopsis pinicola* and the bacterium *Ralstonia pickettii* for DDT biodegradation. Heliyon.

[102] Freddi L., Djokic V., Petot-Bottin F., Girault G., Perrot L., Vicente A.F., Ponsart C. (2021). The use of flocked swabs with a protective medium increases the recovery of live *Brucella* spp. and DNA detection. Microbiol. Spectr..

[103] Chen Y., Chau J., Yoon J., Hladky J. (2022). Rapid, label-free pathogen identification system for multidrug-resistant bacterial wound infection detection on military members in the battlefield. PLoS ONE.

[104] Huang L., Shui X., Wang H., Qiu H., Tao C., Yin H., Wang P. (2023). Effects of *Bacillus halophilus* on growth, intestinal flora and metabolism of *Larimichthys crocea*. Biochem. Biophys. Rep..

[105] Alharbi M.G., Al-Hindi R.R., Alotibi I.A., Azhari S.A., Farsi R.M., Teklemariam A.D. (2023). Evaluation of phage-antibiotic combinations in the treatment of extended-spectrum β-lactamase-producing *Salmonella enteritidis* strain PT1. Heliyon.

[106] Lavigne R., Darius P., Summer E.J., Seto D., Mahadevan P., Nilsson A.S., Ackermann H.W., Kropinski A.M. (2009). Classification of *Myoviridae* bacteriophages using protein sequence similarity. BMC Microbiol..

[107] Uchiyama J., Rashel M., Takemura I., Kato S.-I., Ujihara T., Muraoka A., Matsuzaki S., Daibata M. (2012). Genetic characterization of *Pseudomonas aeruginosa* bacteriophage KPP10. Arch. Virol..

[108] Lohr J.E., Chen F., Hill R.T. (2005). Genomic analysis of bacteriophage ɸJL001: Insights into its interaction with a sponge-associated alpha-proteobacterium. Appl. Environ. Microbiol..

[109] Ceyssens P.-J., Mesyanzhinov V., Sykilinda N., Briers Y., Roucourt B., Lavigne R., Robben J., Domashin A., Miroshnikov K., Volckaert G. (2007). The genome and structural proteome of YuA, a new *Pseudomonas aeruginosa* phage resembling M6. J. Bacteriol..

[110] Dyson Z.A., Seviour R.J., Tucci J., Petrovski S. (2016). Genome sequences of *Pseudomonas oryzihabitans* phage POR1 and *Pseudomonas aeruginosa* phage PAE1. Genome Announc..

[111] Wang X., Tang J., Dang W., Xie Z., Zhang F., Hao X., Sun S., Liu X., Luo Y., Li M. (2023). Isolation and characterization of three *Pseudomonas aeruginosa* viruses with therapeutic potential. Microbiol. Spectr..

[112] Evseev P.V., Gorshkova A.S., Sykilinda N.N., Drucker V.V., Miroshnikov K.A. (2020). *Pseudomonas* bacteriophage AN14—A Baikal-borne representative of *Yuavirus*. Limnol. Freshw. Biol..

[113] Troshin K., Sykilinda N., Shuraleva S., Tokmakova A., Tkachenko N., Kurochkina L., Miroshnikov K., Suzina N., Brzhozovskaya E., Petrova K. (2025). Pseudomonas phage Lydia and the evolution of the *Mesyanzhinovviridae* family. Viruses.

[114] Evseev P., Lukianova A., Sykilinda N., Gorshkova A., Bondar A., Shneider M., Kabilov M., Drucker V., Miroshnikov K. (2021). *Pseudomonas* phage MD8: Genetic mosaicism and challenges of taxonomic classification of lambdoid bacteriophages. Int. J. Mol. Sci..

[115] Burke K.A., Urick C.D., Mzhavia N., Nikolich M.P., Filippov A.A. (2024). Correlation of *Pseudomonas aeruginosa* phage resistance with the numbers and types of antiphage systems. Int. J. Mol. Sci..

[116] Wagemans J., Delattre A.S., Uytterhoeven B., De Smet J., Cenens W., Aertsen A., Ceyssens P.J., Lavigne R. (2015). Antibacterial phage ORFans of *Pseudomonas aeruginosa* phage LUZ24 reveal a novel MvaT inhibiting protein. Front. Microbiol..

[117] Namonyo S., Weynberg K.D., Guo J., Carvalho G. (2023). The effectiveness and role of phages in the disruption and inactivation of clinical *P. aeruginosa* biofilms. Environ. Res..

[118] Kheljan F.S., Hesari F.S., Aminifazl M.S., Skurnik M., Goladze S., Zarrini G. (2023). Design of phage-cocktail-containing hydrogel for the treatment of *Pseudomonas aeruginosa*-infected wounds. Viruses.

[119] Rezk N., Abdelsattar A.S., Elzoghby D., Agwa M.M., Abdelmoteleb M., Aly R.G., Fayez M.S., Essam K., Zaki B.M., El-Shibiny A. (2022). Bacteriophage as a potential therapy to control antibiotic-resistant *Pseudomonas aeruginosa* infection through topical application onto a full-thickness wound in a rat model. J. Genet. Eng. Biotechnol..

[120] Bradley D.E. (1966). The structure and infective process of a *Pseudomonas aeruginosa* bacteriophage containing ribonucleic acid. J. Gen. Microbiol..

[121] Jarrell K., Kropinski A.M. (1977). Identification of the cell wall receptor for bacteriophage E79 in *Pseudomonas aeruginosa* strain PAO. J. Virol..

[122] Ceyssens P.-J., Lavigne R. (2010). Bacteriophages of *Pseudomonas*. Future Microbiol..

[123] Forti F., Bertoli C., Cafora M., Gilardi S., Pistocchi A., Briani F. (2023). Identification and impact on *Pseudomonas aeruginosa* virulence of mutations conferring resistance to a phage cocktail for phage therapy. Microbiol. Spectr..

[124] Markwitz P., Lood C., Olszak T., van Noort V., Lavigne R., Drulis-Kawa Z. (2022). Genome-driven elucidation of phage-host interplay and impact of phage resistance evolution on bacterial fitness. ISME J..

[125] García-Cruz J.C., Rebollar-Juarez X., Limones-Martinez A., Santos-Lopez C.S., Toya S., Maeda T., Ceapă C.D., Blasco L., Tomás M., Díaz-Velásquez C.E. (2024). Resistance against two lytic phage variants attenuates virulence and antibiotic resistance in *Pseudomonas aeruginosa*. Front. Cell. Infect. Microbiol..

[126] Allan B.J., Davies P., Carstens E.B., Kropinski A.M. (1989). Characterization of the genome of *Pseudomonas aeruginosa* bacteriophage ɸPLS27 with particular reference to the ends of the DNA. J. Virol..

[127] Pan X., Cui X., Zhang F., He Y., Li L., Yang H. (2016). Genetic evidence for O-specific antigen as receptor of *Pseudomonas aeruginosa* phage K8 and its genomic analysis. Front. Microbiol..

[128] Ceyssens P.-J., Brabban A., Rogge L., Lewis M.S., Pickard D., Goulding D., Dougan G., Noben J.-P., Kropinski A., Kutter E. (2010). Molecular and physiological analysis of three *Pseudomonas aeruginosa* phages belonging to the “N4-like viruses”. Virology.

[129] Nakayama K., Kanaya S., Ohnishi M., Terawaki Y., Hayashi T. (1999). The complete nucleotide sequence of ɸCTX, a cytotoxin-converting phage of *Pseudomonas aeruginosa*: Implications for phage evolution and horizontal gene transfer via bacteriophages. Mol. Microbiol..

[130] Chibeu A., Ceyssens P.J., Hertveldt K., Volckaert G., Cornelis P., Matthijs S., Lavigne R. (2009). The adsorption of *Pseudomonas aeruginosa* bacteriophage ɸKMV is dependent on expression regulation of type IV pili genes. FEMS Microbiol. Lett..

[131] Yang L., Zhang T., Li L., Zheng C., Tan D., Wu N., Wang M., Zhu T. (2022). Characterization of *Pseudomonas aeruginosa* bacteriophage L5 which requires type IV pili for infection. Front. Microbiol..

[132] Danis-Wlodarczyk K., Vandenheuvel D., Jang H.B., Briers Y., Olszak T., Arabski M., Wasik S., Drabik M., Higgins G., Tyrrell J. (2016). A proposed integrated approach for the preclinical evaluation of phage therapy in *Pseudomonas* infections. Sci. Rep..

[133] Martins L.F., Dos Santos Junior A.P., Nicastro G.G., Scheunemann G., Angeli C.B., Rossi F.P.N., Quaggio R.B., Palmisano G., Sgro G.G., Ishida K. (2024). Phages ZC01 and ZC03 require type-IV pilus for *Pseudomonas aeruginosa* infection and have a potential for therapeutic applications. Microbiol. Spectr..

[134] Olsthoorn R.C., Garde G., Dayhuff T., Atkins J.F., Van Duin J. (1995). Nucleotide sequence of a single-stranded RNA phage from *Pseudomonas aeruginosa*: Kinship to coliphages and conservation of regulatory RNA structures. Virology.

[135] Holland S.J., Sanz C., Perham R.N. (2006). Identification and specificity of pilus adsorption proteins of filamentous bacteriophages infecting *Pseudomonas aeruginosa*. Virology.

[136] Roncero C., Darzins A., Casadaban M.J. (1990). *Pseudomonas aeruginosa* transposable bacteriophages D3112 and B3 require pili and surface growth for adsorption. J. Bacteriol..

[137] Cryz S.J., Pitt T.L., Fürer E., Germanier R. (1984). Role of lipopolysaccharide in virulence of *Pseudomonas aeruginosa*. Infect. Immun..

[138] Comolli J.C., Hauser A.R., Waite L., Whitchurch C.B., Mattick J.S., Engel J.N. (1999). *Pseudomonas aeruginosa* gene products PilT and PilU are required for cytotoxicity in vitro and virulence in a mouse model of acute pneumonia. Infect. Immun..

[139] Jończyk-Matysiak E., Łodej N., Kula D., Owczarek B., Orwat F., Międzybrodzki R., Neuberg J., Bagińska N., Weber-Dąbrowska B., Górski A. (2019). Factors determining phage stability/activity: Challenges in practical phage application. Expert Rev. Anti-Infect. Ther..

[140] Delbruck M. (1945). Interference between bacterial viruses: III. The mutual exclusion effect and the depressor effect. J. Bacteriol..

[141] Bürkle M., Korf I.H.E., Lippegaus A., Krautwurst S., Rohde C., Weissfuss C., Nouailles G., Tene X.M., Gaborieau B., Ghigo G.-M. (2025). Phage-phage competition and biofilms affect interactions between two virulent bacteriophages and *Pseudomonas aeruginosa*. ISME J..

[142] Filippov A.A., Sergueev K.V., He Y., Huang X.Z., Gnade B.T., Mueller A.J., Fernandez-Prada C.M., Nikolich M.P. (2011). Bacteriophage-resistant mutants in *Yersinia pestis*: Identification of phage receptors and attenuation for mice. PLoS ONE.

[143] Mangalea M.R., Duerkop B.A. (2020). Fitness trade-offs resulting from bacteriophage resistance potentiate synergistic antibacterial strategies. Infect. Immun..

[144] Sambrook J., Fritsch E.F., Maniatis T. (1989). Molecular Cloning: A Laboratory Manual.

[145] Bichet M.C., Patwa R., Barr J.J. (2021). Protocols for studying bacteriophage interactions with in vitro epithelial cell layers. STAR Protoc..

[146] Sergueev K.V., Filippov A.A., Farlow J., Su W., Kvachadze L., Balarjishvili N., Kutateladze M., Nikolich M.P. (2019). Correlation of host range expansion of therapeutic bacteriophage Sb-1 with allele state at a hypervariable repeat locus. Appl. Environ. Microbiol..

[147] Sambanthamoorthy K., Gokhale A.A., Lao W., Parashar V., Neiditch M.B., Semmelhack M.F., Lee I., Waters C.M. (2011). Identification of a novel benzimidazole that inhibits bacterial biofilm formation in a broad-spectrum manner. Antimicrob. Agents Chemother..

[148] Ackermann H.W. (2009). Basic phage electron microscopy. Methods Mol. Biol..

[149] Bouras G., Nepal R., Houtak G., Psaltis A.J., Wormald P.-J., Vreugde S. (2023). Pharokka: A fast scalable bacteriophage annotation tool. Bioinformatics.

[150] Camacho C., Coulouris G., Avagyan V., Ma N., Papadopoulos J., Bealer K., Madden T.L. (2009). BLAST+: Architecture and applications. BMC Bioinform..

[151] Meier-Kolthoff J.P., Auch A.F., Klenk H.-P., Göker M. (2013). Genome sequence-based species delimitation with confidence intervals and improved distance functions. BMC Bioinform..

[152] Lefort V., Desper R., Gascue O. (2015). FastME 2.0: A comprehensive, accurate, and fast distance-based phylogeny inference program. Mol. Biol. Evol..

[153] Farris J.S. (1972). Estimating phylogenetic trees from distance matrices. Am. Nat..

[154] Steinegger M., Söding J. (2017). MMseqs2 enables sensitive protein sequence searching for the analysis of massive data sets. Nat. Biotechnol..

[155] Söding J., Biegert A., Lupas A.N. (2005). The HHpred interactive server for protein homology detection and structure prediction. Nucl. Acids Res..

