# Supplementary figures and images for: Pseudomonas aeruginosa Phage Cocktails: Rational Design and Efficacy Against Mouse Wound and Systemic Infection
Source: Antibiotics (Basel). 2026 Jan 9;15(1):75. doi: 10.3390/antibiotics15010075 (PMC12838167; doi:10.3390/antibiotics15010075)

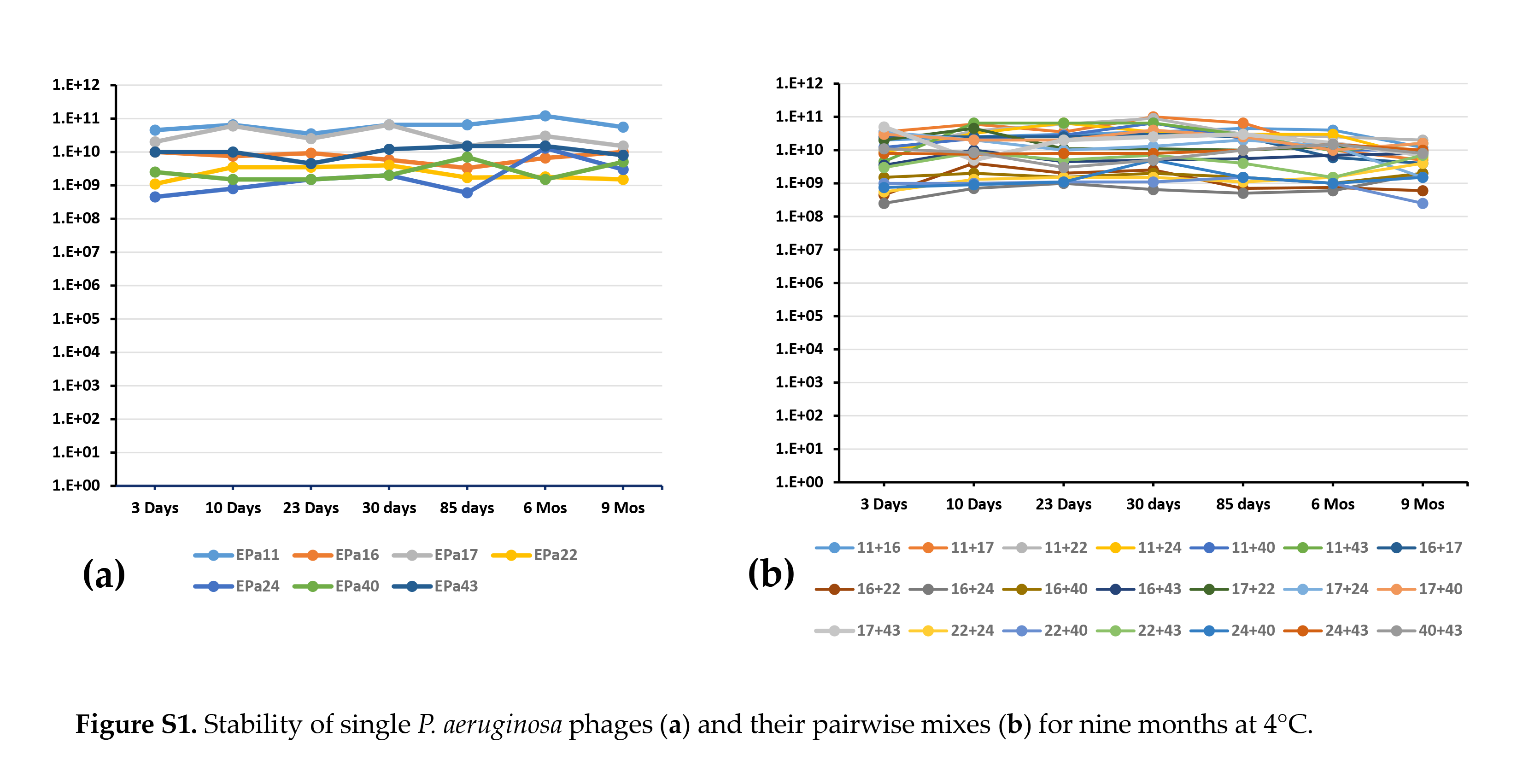

Supplement: Supplementary file 1 [file antibiotics-15-00075-s001.zip › Figure S1.tif]
